# Multivariate Chemometric and FTIR Insights Into the Effects of Rice Bran Extracts on Brown Rice Protein Bars

**DOI:** 10.1002/fsn3.71537

**Published:** 2026-02-17

**Authors:** Zahra Ashrafpour Ardakani, Amir Pourfarzad

**Affiliations:** ^1^ Department of Food Science and Technology, Faculty of Agricultural Sciences University of Guilan Rasht Iran; ^2^ Department of Chemical Technologies Iranian Research Organization for Science & Technology (IROST) Tehran Iran

**Keywords:** antioxidant activity, polyphenolic extract, protein bars, protein secondary structures, rice bran, texture analysis

## Abstract

Protein bars are widely consumed functional snacks, yet their stability and sensory quality can deteriorate during storage. This study examined the impact of incorporating aqueous and ethanolic rice bran extracts—rich sources of phenolic compounds with antioxidant and antimicrobial properties—into brown‐rice‐based protein bars and evaluated their quality, molecular characteristics, and functional performance. Ethanolic extract, which contained 1.85‐fold higher total phenolics than the aqueous extract, produced the most pronounced improvements. Bars fortified with 1% ethanolic extract showed a 37.27% increase in DPPH radical scavenging activity and a 25.74% increase in ferric reducing antioxidant power reducing power compared to the control, alongside a 46.6% reduction in peroxide value, demonstrating enhanced oxidative stability. These molecular effects translated into favorable textural changes, including a 53.63% reduction in hardness and a 28.35% decrease in gumminess, accompanied by an 8.55% improvement in overall sensory acceptability. FTIR spectroscopy demonstrated meaningful alterations in secondary structures, including reduced α‐helix content and increased β‐turn and random coil proportions in ethanolic treatments, indicating partial protein unfolding. These molecular changes corresponded strongly with texture and sensory improvements, as confirmed by multivariate analyses (Principal Component Analysis and Partial Least Squares Regression). Collectively, the results highlight that ethanolic rice bran extract more effectively enhances antioxidant performance, structural properties, and consumer acceptability than aqueous extract. The findings not only underscore its potential as a natural preservative and functional ingredient in high‐protein snacks but also support the valorization of rice bran as a sustainable source of nutraceutical compounds.

## Graphical abstract

Optimal Samples: Ethanolic extract (1%) best overall quality, texture, moisture retention, antimicrobial activity, and sensory scores. Aqueous extract maintained denser protein structure, but slightly lower sensory scores. Conclusion: Ethanolic rice bran extract (1%) improved protein bars in terms of texture, moisture, color, antimicrobial activity, and sensory acceptability. FTIR analysis showed ethanolic extract produced a more flexible protein network, while aqueous extract maintained a denser structure.

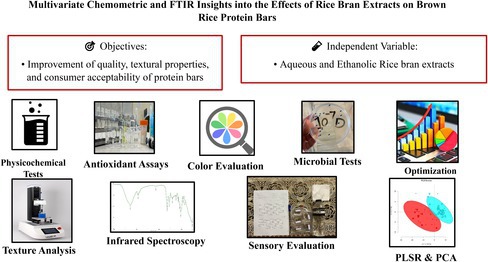

## 1 Introduction

Modern lifestyles and rising health awareness have substantially shaped dietary habits (Thakur et al. 2022). The demand for convenient, nutrient‐dense foods has fueled the expansion of functional products, with protein bars emerging as one of the fastest‐growing categories (Małecki et al. 2022; Szydłowska et al. 2022). First introduced in the 1980s for athletes and vegetarians (Yadav and Bhatnagar 2015), they are now widely consumed as mainstream foods (Małecki et al. 2020). Their popularity stems from high nutritional density, portability, and the absence of preparation requirements (Thakur et al. 2022).

Typically, protein bars contain 20%–50% protein, 20%–50% carbohydrates, and 10%–20% fat (Abdel‐Salam et al. 2022; Hassan 2020). Whey protein is commonly used because of its nutritional value and desirable sensory properties (Małecki et al. 2020). Carbohydrates contribute texture and prevent sugar crystallization (Yadav and Bhatnagar 2015), whereas lipids enhance malleability (Hassan 2020). Brown rice flour is also increasingly used as a functional ingredient because of its bioactive phytochemicals (tocopherols, tocotrienols, γ‐oryzanols, phenolic compounds), fiber, vitamins, and minerals, which contribute to chronic disease prevention (Lee et al. 2019; Saleh et al. 2019; Wu et al. 2017).

A key challenge during storage is bar hardening, which reduces consumer acceptability (Małecki et al. 2022). This is associated with changes in water activity, sugar crystallization, Maillard reactions, and protein aggregation through disulfide bond formation (Hassan 2020; Lu et al. 2016). Since texture and color are critical to consumer preference, mitigating such deterioration is essential (Adámek et al. 2018).

Polyphenolic compounds offer a promising solution. As plant secondary metabolites, they range from phenolic acids to flavonoids and tannins, with strong antioxidant properties (Han et al. 2020; Meng and Li 2021). They not only protect foods from oxidation but also contribute to human health by reducing risks of cancer, type 2 diabetes, and digestive disorders (Han et al. 2020). Cereal grains and their by‐products are valuable natural sources of polyphenols.

Rice bran, the outer layer of brown rice, is particularly rich in polyphenols as well as proteins, fats, vitamins, and minerals (Bhanger et al. 2008; Theerakulkait and Boonsiripiphat 2007). Once used mainly as animal feed, it is now recognized as a functional food ingredient. Rice bran extracts exhibit antioxidant capacity, enhance oxidative stability, and interact with proteins, influencing both structure and functionality (Meng and Li 2021). Traditionally regarded as a low‐value by‐product, it now represents an underutilized resource with high potential for value‐added applications. Recent studies have demonstrated that rice bran can be converted into functional food ingredients, bioactive compounds, and fortifying agents, which not only enhance the nutritional and functional properties of food products but also contribute to waste minimization and circular economy initiatives by transforming milling residues into valuable resources (Nidhishree et al. 2024; Spaggiari et al. 2021). Its incorporation into products such as protein bars, baked goods, and snacks can improve texture, stability, and shelf‐life while simultaneously reducing environmental impact and maximizing resource efficiency (Kaur et al. 2024). Incorporating these extracts into protein bars may therefore help delay hardening, improve texture, and extend shelf life (Diaz et al. 2021).

The extraction method strongly determines the phenolic profile. Organic solvent maceration remains common (Ruen‐Ngam et al. 2014). Although methanol was once effective, its toxicity limits food applications (Tabaraki and Nateghi 2011). Ethanol, a safe and polar solvent, extracts a wide range of phenolics with high efficiency (Peanparkdee and Iwamoto 2019). Water is another conventional, eco‐friendly solvent (Sukhonthara et al. 2016). Thus, both ethanolic and aqueous rice bran extracts are suitable candidates for food fortification.

Although plant extracts have been broadly investigated as natural antioxidants and stabilizing agents in various food matrices (Salitlertthanasin 2017; Showkat et al. 2018), their use in protein bar systems—particularly extracts derived from rice bran—remains unexplored. Given that rice bran is a nutritionally rich yet underutilized by‐product, its valorization aligns with current global priorities in sustainable food formulation and circular economy strategies. Therefore, this study evaluates how aqueous and ethanolic rice bran extracts modulate the physicochemical, antioxidant, microbial, sensory, and protein secondary structural properties of brown‐rice‐based protein bars. Beyond conventional functional assessments, the work uniquely integrates FTIR‐derived secondary structural profiling with chemometric tools [principal component analysis (PCA) and partial least squares regression (PLSR)] to unravel mechanistic linkages between polyphenol–protein interactions, textural evolution, and sensory outcomes. This combined analytical–multivariate framework distinguishes the present study from previous research on protein bars and provides a novel, optimization‐oriented perspective that has not been reported for any plant‐based protein bar fortified with rice bran extract.

## 2 Materials and Methods

### 2.1 Materials

Stabilized rice bran and stabilized brown rice flour were purchased from Giltaz Company (Guilan, Iran). According to the manufacturer, the flour contained approximately 8.7% moisture, 14.6% protein, 15.4% fat, 1.2% ash, 9.4% crude fiber, and 35.22 mg/100 g phytic acid. Whey protein concentrate (WPC) was procured from Ruysa Shop (Mazandaran, Iran), containing approximately 80% protein, 6%–8% lactose, 5%–7% fat, and 2%–4% moisture, as specified by the manufacturer. Additional ingredients included glycerol (food‐grade; Arian Glycerin Co., Iran) as a humectant and plasticizer, refined sunflower oil (food‐grade; Behshahr Co., Iran) as a lipid source, and distilled water for hydration. All ingredients were of food‐grade quality and used without further purification.

### 2.2 Methods

#### 2.2.1 Preparation of Aqueous and Ethanolic Rice Bran Extracts

Aqueous and ethanolic rice bran extracts were prepared at Giah‐Kala Company (Sabzevar, Iran) using the maceration method (Surarit et al. 2015). Stabilized rice bran was sieved, and 25 g was mixed with 100 mL of either distilled water or absolute ethanol. The mixture was placed on an electric shaker at room temperature for 3 h. This extraction was repeated twice more with 100 mL of the same solvent containing 0.15% HCl under identical conditions. The resulting extracts were combined, filtered through a 0.45 μm nylon membrane, and concentrated using a rotary evaporator at 45°C. The concentrated extracts were stored at 4°C until further use.

#### 2.2.2 Preparation of Protein Bars

Protein bars were prepared using a cold‐processing method at 25°C to prevent the degradation of heat‐sensitive compounds, such as whey proteins and bioactive components in rice bran extracts. The optimized formulation consisted of 38.66% whey protein, 19.33% brown rice flour, 9.80% sunflower oil, 16.01% food‐grade glycerol, and 16.20% distilled water.

First, the dry ingredients were mixed at 200 rpm for 3 min to achieve preliminary homogeneity. Then, the liquid ingredients—including aqueous and ethanolic rice bran extracts, water, glycerol, and sunflower oil—were gradually added to the dry mixture and homogenized at 50 rpm for 5 min. This process minimized air incorporation and ensured a uniform texture without undesirable voids.

The final mixture was poured into silicone molds (2 × 2 × 2 cm) to produce samples of uniform size and shape for analytical testing. Samples were stored in closed containers at 25°C for 7 days to reach moisture equilibrium. Different levels of aqueous and ethanolic rice bran extracts were incorporated according to the experimental design. The extract levels (0.5%–2%) were calculated on the basis of the total wet weight of the protein bars. Considering the dry matter content of the extracts, the corresponding amount of water in the formulation was adjusted to maintain consistent texture and composition. This method preserved the native protein structure by avoiding heat application and thus maintained the functional, nutritional, and sensory properties of the product. Component ratios were optimized on the basis of preliminary trials to achieve a balanced texture, moisture content, structural integrity, and sensory acceptance (Movaghar, Pourfarzad, & Mehregan Nikoo, 2025; Pourghasemian et al. 2025b; Talemi et al. 2025).

#### 2.2.3 Proximate Composition Analysis of Protein Bars

The proximate composition (moisture, ash, crude lipids, and crude protein) of protein bars formulated with aqueous and ethanolic rice bran extracts and rice bran flour was determined according to AOAC methods. Crude protein (*N* × 6.25) was measured using the Kjeldahl method (Samuel and Peerkhan 2020; Szydłowska et al. 2022). Moisture content was determined by oven‐drying the samples at 105°C until constant weight, whereas ash content was measured after incineration at 550°C. Fat content was analyzed by acid hydrolysis followed by petroleum ether extraction using a Soxhlet apparatus (Samuel and Peerkhan 2020; Torres et al. 2011). Crude carbohydrate content was calculated by difference, subtracting protein, lipids, moisture, and ash values (Szydłowska et al. 2022). The energy value was estimated using the Atwater general factor system: 4 kcal/g for protein, 9 kcal/g for fat, and 4 kcal/g for carbohydrates (Abdel‐Salam et al. 2022).

#### 2.2.4 Water Activity

Water activity was determined using a water activity meter. Approximately 5 g of each sample was placed in the test container and inserted into the measuring chamber. The final value for each sample was calculated as the mean of three replicates (Małecki et al. 2022).

#### 2.2.5 Measurement of pH

The pH of each sample was measured in triplicate using a calibrated digital pH meter (Abdel‐Salam et al. 2022). For this purpose, 2.5 g of the homogenized sample was mixed with 25 mL of freshly boiled distilled water and allowed to settle for 20 min before measurement.

#### 2.2.6 Acidity

Five grams of the sample were dispersed in 50 mL of distilled water and mixed thoroughly. The mixture was allowed to stand for 1 h, after which 0.5 mL of phenolphthalein indicator was added. The solution was titrated with standardized 0.1 N NaOH until a faint pink color persisted for 30 s (Turgut et al. 2014).

#### 2.2.7 Peroxide Value

Four grams of the sample (M) were mixed with 10 mL of chloroform and 15 mL of glacial acetic acid. After 30 min, the mixture was filtered using qualitative filter paper (125 mm). The filtrate was vigorously shaken with 1 mL of saturated aqueous potassium iodide (KI) solution added dropwise, and the sample was kept in the dark for 5 min. An equal volume of distilled water was then added, and the liberated iodine was titrated with 0.01 M sodium thiosulfate solution (T). A blank (V0) without sample was also prepared. The peroxide value (PV) was calculated as milliequivalents of active oxygen per kilogram of sample (mEq O_2_/kg) using the equation:

(1) $$\mathrm{PV}=\frac{\left(V_{1}-V_{0}\right)T\times 10^{3}}{M}$$

where *V*_1_ = volume of sodium thiosulfate used (mL), *V*_0_ = blank volume (mL), T = molarity of sodium thiosulfate, and *M* = mass of sample (g) (Okpala et al. 2016)

#### 2.2.8 Antioxidant Activity

##### 2.2.8.1 Extract Preparation

Extracts were prepared by mixing 25 g of crushed protein bar with 100 mL of ethanol. The mixture was shaken and stored in the dark at room temperature for 20 h and then filtered (Szydłowska et al. 2020).

##### 2.2.8.2 Total Phenolic Content (TPC) Determination

An aliquot of 0.2 mL extract was mixed with 0.8 mL Folin–Ciocalteu reagent. An ethanol/water mixture (50:50, v/v) was used as the blank. After 2 min, 2 mL of 7.5% (w/v) sodium carbonate solution was added, and the volume was adjusted to 10 mL with distilled water. The mixture was kept in the dark for 60 min, and absorbance was measured at 725 nm (Irakli et al. 2018; Peanparkdee et al. 2019).

##### 2.2.8.3 Ferric Reducing Antioxidant Power (FRAP)

One milliliter of extract was combined with 2.5 mL of 0.1 M phosphate buffer (pH 6.6) and 2.5 mL of 1% (w/v) potassium ferricyanide solution. The mixture was incubated in a water bath at 50°C for 20 min, followed by the addition of 2.5 mL of 10% (w/v) trichloroacetic acid. From this, 2.5 mL was taken, mixed with 2.5 mL distilled water and 0.5 mL of 0.1% (w/v) ferric chloride. After 30 min, absorbance was measured at 700 nm (Abdel‐Salam et al. 2022).

##### 2.2.8.4 DPPH Assay

A 0.1 mM ethanolic DPPH solution was prepared. Then, 0.2 mL of extract was mixed with 3 mL of DPPH solution and kept in the dark for 30 min. Absorbance was measured at 517 nm, and radical scavenging activity (RSA, %) was calculated using (Baliyan et al. 2022):

(2) $$\%\mathrm{RSA}=\frac{\left(A_{\text{control}}-A_{\text{sample}}\right)\times 100}{A_{\text{control}}}$$

##### 2.2.8.5 Determination of TFC

An aliquot of 0.5 mL extract was mixed with 2.5 mL distilled water and 0.15 mL of 5% NaNO_2_ solution. After 6 min, 0.3 mL of 10% AlCl_3_.6H_2_O solution was added. After 5 min, 1.0 mL of 0.1 M NaOH and 0.55 mL of distilled water were added. Absorbance was measured immediately at 370 nm (Wanyo et al. 2014).

#### 2.2.9 Microbiological Quality

Twenty‐five grams of the sample was homogenized with 225 mL of peptone water and serially diluted. Aliquots were surface‐spread on duplicate plates of the appropriate media. Total viable counts were determined on nutrient agar after incubation at 37°C for 48 h. Molds and yeasts were enumerated on YGC agar incubated at 25°C for 120 h (Szydłowska et al. 2020; Trząskowska et al. 2022).

#### 2.2.10 Texture Profile Analysis (TPA)

TPA of the brown rice protein bars was performed using a Brookfield CT3‐10 K texture analyzer (Brookfield Engineering Laboratories, Massachusetts, USA). A cylindrical probe (TA4/1000, 38.11 mm diameter) with a 10 kg load cell was used to compress the samples to 30% deformation at a crosshead speed of 5 mm/s. The parameters measured included hardness (N), adhesiveness, cohesiveness, gumminess (N), springiness, and chewiness (N) (Jabeen et al. 2020; Małecki et al. 2020).

#### 2.2.11 Color Measurement

Color was analyzed using image processing. RGB images were captured against a white background in JPEG format and processed with ImageJ software. Images were converted to CIELab coordinates, and L*, a*, and b* values were calculated (Srisuk and Jirasatid 2023). The total color difference (ΔE) between fresh and stored samples was calculated using the Hunter–Scotfield equation:

(3) $$\mathrm{\Delta E}=\sqrt{{\left(L_{0}-L\right)}^{2}+{\left(a_{0}-a\right)}^{2}+{\left(b_{0}-b\right)}^{2}}$$

where *L*_0_*, *a*_0_*, and *b*_0_* are initial values, and L*, a*, and b* are measured values. Browning index (BI), related to enzymatic and non‐enzymatic browning, was calculated as:

(4) $$\mathrm{BI}=\frac{100\left(x-0.31\right)}{0.172}$$

(5) $$x=\frac{\left(a^{*}+1.75L^{*}\right)}{\left(5.645L^{*}+a^{*}-3.012b^{*}\right)}$$

#### 2.2.12 FTIR Spectroscopy Analysis

Protein structural changes were analyzed using FTIR (Jasco 4700, Japan) in ATR mode. Spectra were collected over the range of 4000–400 cm^−1^ with a resolution of 4 cm^−1^. Prior to analysis, all spectra were thoroughly preprocessed: baseline correction was performed to eliminate background drift, and vector normalization (OMNIC software) was applied to ensure comparability of spectral intensities across samples. The amide I region (1600–1700 cm^−1^) was refined through region‐specific baseline correction to enhance component separation. Second‐derivative transformation was then used to resolve overlapping peaks, followed by Gaussian curve fitting in PeakFit to quantify individual secondary structure components, including α‐helix, intra‐ and intermolecular β‐sheets, β‐turns, and random coils. These standardized preprocessing and deconvolution procedures ensured accurate and reproducible characterization of protein structural changes (Pourfarzad et al. 2019, 2015).

#### 2.2.13 Sensory Evaluation

Sensory evaluation was performed by a trained panel consisting of 10 adult members (aged 23–45 years; 6 females and 4 males) with prior experience in quality assessment of cereal‐ and protein‐based products. Approximately 10 g of each bar was served in coded (three‐digit) plastic containers at room temperature. The evaluation was conducted under blind and randomized conditions using a fully randomized serving order to minimize bias. Panelists were instructed to rinse their mouths with water between samples.

The sensory attributes evaluated included color, taste, odor, texture, chewiness, flavor, and overall acceptability, using a 5‐point hedonic scale (1 = extremely dislike, 5 = extremely like) (Małecki et al. 2020). All participants provided written informed consent prior to testing. According to institutional ethical guidelines for minimal‐risk sensory studies involving trained adult assessors, no separate ethics approval code was required; however, the study adhered fully to institutional ethical standards, as stated in the Ethical Approval section.

#### 2.2.14 Statistical Analysis and Optimization

Experiments were conducted using a completely randomized design with three replications. All results are expressed as mean ± standard deviation (*n* = 3). Data related to physicochemical, antioxidant, microbial, color, sensory, and FTIR‐based protein structural properties were analyzed using one‐way ANOVA, and mean comparisons were performed with Duncan's multiple range test at *p* < 0.05 (Minitab 21.3, Minitab Inc., State College, PA, USA).

Multi‐response optimization was carried out using the desirability function approach. For each response variable, a specific goal (maximize, minimize, or in‐range), weight, and importance level (1–5) were assigned on the basis of its contribution to overall product quality. Desirability values (*d*ᵢ) were computed by transforming each response into a scale from 0 (undesirable) to 1 (fully desirable). The overall desirability (D) was calculated as the geometric mean of all individual desirabilities.

Attributes such as hardness, chewiness, peroxide value, BI, aw, acidity, and microbial counts were defined as minimized; sensory attributes, antioxidant activities (TPC, TFC, FRAP, and DPPH), textural positivity indices (cohesiveness and springiness), protein content, and FTIR‐derived structures (β‐turn, intra‐β‐sheet, and random coil) were defined as maximized. Color parameters with acceptable variation (a*, b*, pH, and ash) were set as in‐range.

The optimization produced the formulation with the highest overall desirability value, ensuring a balanced enhancement of physicochemical, antioxidant, structural, sensory, and textural attributes. The complete optimization criteria—including goals, weights, and importance scores—are provided in Table 1.

**TABLE 1:** Goals, weights, and importance values used for multi‐response optimization.

Attribute	Weight	Importance	Goal
Moisture content (%)	5	3	Minimize
Ash content (%)	4	2	In range
Protein content (%)	10	5	Maximize
Fat content (%)	4	2	Minimize
Carbohydrate content (%)	4	2	Minimize
Total energy (kcal/100 g)	8	4	Maximize
Water activity, aw	8	4	Minimize
pH	4	2	In range
Titratable acidity (g citric acid/100 g)	5	3	Minimize
Peroxide value (meq O_2_/kg oil)	8	4	Minimize
Total polyphenols (mg GAE/g)	10	5	Maximize
FRAP (μmol Fe^2+^/g extract)	10	5	Maximize
DPPH scavenging activity (%)	10	5	Maximize
Total flavonoids (mg QE/g)	10	5	Maximize
Total viable count, TVC (log CFU/g)	8	4	Minimize
Total yeast and mold count, TYMC (log CFU/g)	8	4	Minimize
Hardness (N)	10	5	Minimize
Adhesiveness (N·s)	7	4	Minimize
Cohesiveness	7	4	Maximize
Springiness (mm)	6	3	Maximize
Gumminess (N)	6	3	Maximize
Chewiness (N·mm)	7	4	Minimize
L*	8	4	Maximize
a*	5	3	In range
b*	5	3	In range
∆E	8	4	Minimize
Browning index (BI)	10	5	Minimize
α‐Helix (relative percentage, %)	10	5	Minimize
β‐Turn (relative percentage, %)	10	5	Maximize
Inter‐molecular β‐sheet (relative percentage, %)	10	5	Minimize
Intra‐molecular β‐sheet (relative percentage, %)	10	5	Maximize
Random coil (relative percentage, %)	10	5	Maximize
Color (sensory score, 1–5)	8	4	Maximize
Odor (sensory score, 1–5)	5	3	Maximize
Texture (sensory score, 1–5)	10	5	Maximize
Flavor (sensory score, 1–5)	5	3	Maximize
Chewiness (sensory score, 1–5)	7	4	Maximize
Overall acceptability (sensory score, 1–5)	10	5	Maximize

PCA and PLSR were applied to the mean‐centered datasets of physicochemical properties, FTIR‐derived secondary structures, and sensory attributes to investigate correlations and identify predictors of overall quality performance.

## 3 Results and Discussion

### 3.1 Analyses of Aqueous and Ethanolic Rice Bran Extracts

The proximate composition of the aqueous and ethanolic rice bran extracts is presented in Table 2, showing significant differences depending on the solvent used. Moisture was higher in the aqueous extract (79.0% FW) than in the ethanolic extract (30.94% FW), which corresponded with higher °Brix values (Huang et al. 2024). Protein and carbohydrate contents were also greater in the aqueous extract (13.24% and 77.52% DW, respectively) compared to the ethanolic extract (9.82% and 76.14% DW), whereas ash content was higher in the ethanolic extract (8.42% DW) than in the aqueous extract (6.05% DW). The pH values of both extracts were comparable (4.65 vs. 4.64). These differences are consistent with the polarity of the solvents: water preferentially extracts polar constituents such as proteins and carbohydrates, whereas ethanol is more effective in recovering certain minerals and lipophilic compounds (Jin et al. 2023; Usman et al. 2023).

**TABLE 2:** Nutritional and functional characterization of aqueous and ethanolic rice bran extracts.

Composition	Aqueous extract	Ethanolic extract
Moisture (% wet weight)	79.00 ± 0.009	94.30 ± 0.002
Ash (% Dry weight)	6.05 ± 0.001	8.42 ± 0.001
Fat (% Dry weight)	3.21 ± 0.001	5.61 ± 0.001
Protein (% Dry weight)	13.24 ± 0.002	9.82 ± 0.001
Carbohydrate (% Dry weight)	52.77 ± 0.001	76.14 ± 0.001
pH	65.4 ± 0.01	4.64 ± 0.01
TPC (mg GAE/g dry weight)	53.29 ± 0.067	68.85 ± 0.021
TFC (mg QE/g dry weight)	15.99 ± 0.004	20.66 ± 0.006
DPPH radical scavenging (%)	60.04 ± 0.112	81.17 ± 0.444
FRAP (μmol Fe^2+^/g dry weight)	65.14 ± 0.043	70.28 ± 0.002

*Note:* means ± standard deviation (*n* = 3).

Regarding bioactive compounds, the TPC was markedly higher in the ethanolic extract (68.85 mg GAE/g DW) compared to the aqueous extract (53.29 mg GAE/g DW). This difference is attributed to the semi‐polar nature of ethanol, which enables the extraction of both free and cell wall‐bound phenolics, thereby broadening the range of recovered compounds (Mandal et al. 2023; Seneviratne et al. 2022). Similarly, the total flavonoid content (TFC) was greater in the ethanolic extract (20.66 mg quercetin equivalents (QE)/g DW) than in the aqueous extract (15.99 mg QE/g DW). This finding reflects the moderate‐to‐low polarity of most flavonoids and the superior penetration ability of ethanol, which facilitates the solubilization of lipophilic flavonoids bound within the rice bran cell wall matrix.

In terms of antioxidant capacity, the ethanolic extract exhibited stronger radical scavenging and reducing activities. Its DPPH radical scavenging activity (81.17%) was considerably higher than that of the aqueous extract (60.04%), in agreement with the higher TPC and extraction efficiency of ethanol (Das et al. 2018; Ghasemzadeh et al. 2018). Likewise, the FRAP was greater in the ethanolic extract, indicating enhanced electron‐donating capacity. This result highlights the key role of phenolic and flavonoid compounds—more effectively extracted by ethanol—in contributing to the reducing activity and overall antioxidant potential of rice bran extracts (Seneviratne et al. 2022).

### 3.2 Physicochemical Composition of Protein Bars

The proximate composition of the protein bars, including protein, moisture, ash, fat, total carbohydrate, and energy is presented in Table 3.

**TABLE 3:** Chemical composition of protein bars supplemented with different levels of rice bran extracts.

Treatment		Moisture (%)	Protein (%)	Fat (%)	Ash (%)	Carbohydrate (%)	Energy (Kcal/100 g)
Control		15.96 ± 0.003^d^	33.76 ± 0.015^a^	11.63 ± 0.005^a^	1.92 ± 0.003^a^	52.70 ± 0.020^b^	450.50 ± 0.030^a^
Aqueous	0.50%	17.30 ± 0.003^b^	33.46 ± 0.014^a^	11.58 ± 0.005^a^	1.94 ± 0.004^a^	52.85 ± 0.020^b^	450.10 ± 0.030^a^
	1%	17.00 ± 0.001^bc^	33.45 ± 0.013^a^	11.50 ± 0.006^a^	1.96 ± 0.004^a^	53.09 ± 0.020^b^	449.70 ± 0.040^a^
	1.50%	16.46 ± 0.003^cd^	33.20 ± 0.012^b^	11.41 ± 0.007^a^	1.98 ± 0.003^a^	53.41 ± 0.020^ab^	449.10 ± 0.040^a^
	2%	16.16 ± 0.001^d^	32.89 ± 0.012^b^	11.30 ± 0.007^b^	1.99 ± 0.003^a^	53.82 ± 0.030^a^	448.50 ± 0.040^b^
Ethanolic	0.50%	17.03 ± 0.004^bc^	33.62 ± 0.014^a^	11.59 ± 0.005^a^	1.94 ± 0.004^a^	52.85 ± 0.020^b^	450.20 ± 0.030^a^
	1%	17.36 ± 0.003^b^	33.48 ± 0.013^a^	11.56 ± 0.006^a^	1.96 ± 0.004^a^	53.00 ± 0.020^b^	450.0 ± 0.030^a^
	1.50%	17.73 ± 0.001^ab^	33.35 ± 0.012^b^	11.52 ± 0.006^a^	1.98 ± 0.003^a^	53.14 ± 0.020^b^	440.70 ± 0.040^a^
	2%	18.30 ± 0.002^a^	33.21 ± 0.013^b^	11.49 ± 0.001^a^	2.00 ± 0.013^a^	53.29 ± 0.020^b^	449.40 ± 0.040^b^

*Note:* Values are presented as mean ± standard deviation (*n* = 3). Different superscript letters within the same column indicate significant differences among treatments at 5% significance level.

#### 3.2.1 Moisture

Moisture content is a key factor influencing shelf‐life, texture, taste, and consumer acceptability (Szydłowska et al. 2022). The control sample (without extract) had the lowest value (15.97%), whereas bars containing aqueous extract (0.5%–2%) ranged from 16.17% to 17.30%, and those with ethanolic extract ranged from 17.03% to 18.30%.

Although initial formulations were moisture‐adjusted, final values after 7 days of storage were significantly influenced by extract type and concentration. Ethanolic extract, in particular, caused a dose‐dependent increase in moisture retention. This effect is likely due to polyphenols and flavonoids forming hydrogen bonds with water, thereby reducing evaporation during storage (Diaz et al. 2021; Meng and Li 2021; Xue et al. 2024). Interestingly, at higher aqueous extract levels, a slight decrease in moisture was noted, which may be linked to matrix compaction and facilitated water loss. These findings suggest that bioactive compounds exert dual effects on water retention depending on extract type and concentration (Alba et al. 2019; Świeca et al. 2017).

#### 3.2.2 Protein

Protein is a vital nutrient with both functional and nutritional roles, essential for growth, skeletal development, and muscle maintenance (Lu et al. 2016; Tasnim et al. 2020). Recommended intakes range from 0.8 g/kg/day in healthy adults to 1.2–2 g/kg/day in athletes (Jäger et al. 2017).

In this study, the control bars contained 33.76 g protein/100 g. Bars with aqueous extract ranged between 32.89% and 33.64%, whereas ethanolic extract bars contained 33.21%–33.62%. Protein reduction in supplemented bars was minor but more evident at higher extract levels. This may result from dilution by non‐protein components of the extracts and potential interactions of polyphenols with protein amino groups, which could alter conformation and lower apparent Kjeldahl values (Nemli et al. 2024).

#### 3.2.3 Fat

Fat provides energy and supports muscle performance, with dietary recommendations suggesting 20%–35% of caloric intake from fat (Burke et al. 2013).

Fat levels in the bars ranged from 11.49% to 11.63%, with no significant differences across treatments. The extracts themselves are nearly fat‐free, and their inclusion did not replace fat sources in the formulation. Thus, extract addition did not affect the nutritional or functional fat‐related attributes of the bars (Tan et al. 2023).

#### 3.2.4 Ash

Ash represents the mineral fraction of foods (Abdel‐Salam et al. 2022; Harris and Marshall 2017). The ash content of the bars ranged from 1.92% to 2.00%, with no significant treatment differences. This stability reflects both the low mineral content of the extracts and their limited impact at the tested concentrations (Bhosale and Vijayalakshmi 2015).

#### 3.2.5 Carbohydrate

Carbohydrates are essential energy substrates for muscles and the nervous system (Burke et al. 2013; Sunyoto et al. 2019). In the current formulation, protein and fat were the primary caloric sources, with carbohydrates contributing less (Samuel and Peerkhan 2020).

Extract supplementation did not significantly affect carbohydrate content, except for slight increases in bars containing 1.5%–2% aqueous extract. This was likely due to water‐soluble carbohydrates introduced with the extracts (Antunes et al. 2023; Eng and Mohd Rozalli 2022).

#### 3.2.6 Energy

Energy content, derived from protein, fat, and carbohydrate contributions, is a critical dietary parameter (Abdel‐Salam et al. 2022; Harwood and Drake 2019). Average daily needs are 2200 kcal for women, 3000 kcal for men, and 40–70 kcal/kg for athletes (Umme et al. 2021).

In this study, the bars provided up to 450 kcal/100 g. The lowest values were seen in bars containing 2% extract (aqueous or ethanolic). Overall, extract supplementation had negligible effects on caloric value, maintaining the bars' suitability as energy‐dense snacks or meal replacements (Gul et al. 2015; Szydłowska et al. 2022).

### 3.3 Water Activity

Water activity (a_w_), defined as the ratio of the vapor pressure of water in a food to that of pure water under identical conditions, reflects the availability of water for microbial growth and biochemical processes (Małecki et al. 2022). In protein bars, a_w_ is affected by the type and amount of proteins, carbohydrates, and fats (Hassan 2020).

In this study, a_w_ values ranged from 0.779 to 0.792 in bars with aqueous rice bran extract and 0.786–0.796 in those with ethanolic extract (Table 4). Extract addition slightly decreased a_w_ compared to the control. This reduction is attributed to the presence of glycerol, which lowers aw by retaining water in the protein matrix (Hassan 2020; Srebernich et al. 2016), as well as to polyphenols forming insoluble complexes with proteins, thereby restricting water mobility (Adrar et al. 2019; Diaz et al. 2021; Xue et al. 2024). Lower a_w_ in bars with aqueous extract may further reflect its higher starch and fiber content, which can bind water and create dense networks that limit free water (Alba et al. 2019). These results suggest that rice bran extracts reduce aw and may contribute to improved microbial stability of protein bars.

**TABLE 4:** Physicochemical properties of protein bars supplemented with different levels of rice bran extracts.

Treatment		Water activity	pH	Titratable acidity (%)	Peroxide value (meq O_2_/kg)
Control		0.801 ± 0.005^a^	6.55 ± 0.015^a^	1.00 ± 0.000^a^	2.48 ± 0.000^a^
Aqueous	0.50%	0.792 ± 0.000^c^	6.55 ± 0.010^a^	1.00 ± 0.001^a^	2.24 ± 0.004^b^
	1%	0.790 ± 0.001^c^	6.54 ± 0.005^ab^	1.00 ± 0.001^a^	1.74 ± 0.002^d^
	1.50%	0.787 ± 0.001^d^	6.53 ± 0.010^bc^	1.00 ± 0.001^a^	1.36 ± 0.171^e^
	2%	0.779 ± 0.001^e^	6.52 ± 0.010^c^	1.00 ± 0.001^a^	0.73 ± 0.006^h^
Ethanolic	0.50%	0.796 ± 0.002^b^	6.55 ± 0.010^a^	1.1 ± 0.001^a^	1.86 ± 0.179^c^
	1%	0.790 ± 0.001^c^	6.54 ± 0.010^ab^	1.00 ± 0.001^a^	1.32 ± 0.119^f^
	1.50%	0.790 ± 0.001^c^	6.52 ± 0.010^c^	1.00 ± 0.001^a^	0.74 ± 0.005^g^
	2%	0.786 ± 0.001^d^	6.51 ± 0.010^c^	1.00 ± 0.001^a^	0.49 ± 0/004^a^

*Note:* Values are presented as mean ± standard deviation (*n* = 3). Different superscript letters within the same column indicate significant differences among treatments at 5% significance level.

### 3.4 pH

pH is a critical factor affecting protein functionality, emulsification, gelation, and overall stability of foods (Karastogianni et al. 2016).

Here, only minor variations were observed: the control sample had a pH of 6.55, whereas bars with aqueous and ethanolic extracts ranged between 6.51 and 6.55. This slight acidity likely results from polyphenols and organic acids that act as weak acids (Ishak et al. 2019). Thus, incorporation of rice bran extracts did not significantly alter pH, ensuring near‐neutral conditions favorable for both product stability and consumer acceptability.

### 3.5 Titratable Acidity

Titratable acidity (TA) reflects the total content of organic acids that influence flavor and stability (Tyl and Sadler 2017).

In this study, the addition of aqueous or ethanolic extracts had no significant effect on TA, which agrees with previous reports on rice bran extracts in probiotic beverages (Hatami et al. 2023). The lack of change in TA further confirms the stability of extract‐fortified protein bars in terms of acidity‐related quality attributes.

### 3.6 Peroxide Value

Peroxide value (PV) indicates the extent of primary lipid oxidation (Bhanger et al. 2008; Okpala et al. 2016).

The control sample showed the highest PV (2.49 meq O_2_/kg), whereas bars with aqueous and ethanolic extracts ranged from 0.73 to 2.24 and 0.50–1.87 meq O_2_/kg, respectively (Table 4). Increasing extract concentration significantly reduced PV, reflecting the antioxidant capacity of phenolics and flavonoids that inhibit lipid peroxidation. Ethanolic extract was more effective than aqueous extract because of its higher content of lipophilic antioxidants. These findings highlight the potential of rice bran extracts, particularly ethanolic ones, as natural antioxidants to improve lipid stability and shelf life of protein bars.

### 3.7 Antioxidant Properties

The antioxidant potential of the protein bars was evaluated through TPC, TFC, DPPH radical scavenging activity, and FRAP assay (Table 5).

**TABLE 5:** Antioxidant properties of protein bars supplemented with different levels of rice bran extracts.

Treatment		TPC (mg GAE/g)	TFC (mg QE/g)	DPPH (%)	FRAP (μmol Fe^2+^/g)
Control		1.58 ± 0.004^f^	1.24 ± 0.003^e^	45.17 ± 0.104^i^	36.72 ± 0.120^i^
Aqueous	0.50%	1.89 ± 0.005^e^	1.33 ± 0.004^d^	59.34 ± 0.419^h^	39.33 ± 0.12^h^
	1%	2.19 ± 0.005^d^	1.41 ± 0.005^c^	62.01 ± 0.209^g^	45.79 ± 0.24^g^
	1.50%	2.49 ± 0.006^c^	1.49 ± 0.005^b^	64.16 ± 0.314^f^	50.08 ± 0.12^f^
	2%	2.78 ± 0.006^b^	1.57 ± 0.006^a^	65.43 ± 0.209^e^	53.14 ± 0.49^e^
Ethanolic	0.50%	1.98 ± 0.005^e^	1.35 ± 0.004^c^	69.71 ± 0.442^d^	56.83 ± 0.61^d^
	1%	2.38 ± 0.005^c^	1.47 ± 0.005^b^	72.55 ± 0.209^c^	59.57 ± 0.37^c^
	1.50%	2.78 ± 0.006^b^	1.58 ± 0.006^a^	75.81 ± 0.419^b^	66.97 ± 0.24^b^
	2%	3.18 ± 0.006^a^	1.70 ± 0.006^a^	78.93 ± 0.209^a^	76.28 ± 0.49^a^

*Note:* Values are presented as mean ± standard deviation (*n* = 3). Different superscript letters within the same column indicate significant differences among treatments at 5% significance level.

#### 3.7.1 Total Polyphenol Content

Control bars contained baseline levels of polyphenols derived from brown rice flour (Saleh et al. 2019). The addition of rice bran extracts significantly elevated TPC in all treatments, with ethanolic extract producing the highest values. This difference reflects the intermediate polarity of ethanol, which allows efficient solubilization of both free and bound polyphenols, including lipophilic fractions less accessible to water extraction (Bhat & Riar, 2017). A clear dose‐dependent increase in TPC was observed, highlighting the extract concentration as a key determinant of antioxidant fortification. These findings are consistent with prior studies reporting ethanol as the superior solvent for phenolic recovery from cereals (Ghasemzadeh et al. 2015; Martillanes, Rocha‐Pimienta, et al. 2020).

#### 3.7.2 Total Flavonoid Content

Flavonoids, as an important subclass of polyphenols, were significantly fortified in bars containing rice bran extracts. Ethanolic extract again yielded higher values than aqueous extract, consistent with the medium‐to‐low polarity of many flavonoids that limits their solubility in water but favors ethanol (Peanparkdee and Iwamoto 2019). The observed increase not only confirms successful incorporation of bioactive compounds into the protein bar matrix but also indicates that flavonoids are effectively stabilized within the bar system. This stabilization may enhance their bioavailability upon consumption.

#### 3.7.3 DPPH Radical Scavenging Activity

Antioxidant capacity measured by DPPH assay increased significantly with rice bran extract addition. Control bars exhibited the lowest activity, attributable only to natural antioxidants present in whey protein and rice flour. In contrast, ethanolic extract treatments displayed superior radical scavenging capacity, reflecting their higher phenolic and flavonoid content and the ability of ethanol to extract diverse antioxidant molecules with varying polarity (Andriani et al. 2022). The results suggest that antioxidant compounds not only survived processing but also retained functional activity, supporting the feasibility of protein bars as carriers of bioactive ingredients.

#### 3.7.4 Ferric Reducing Antioxidant Power

FRAP results followed a pattern similar to DPPH, with the control showing the lowest reducing capacity and bars containing 2% ethanolic extract achieving the highest levels. The strong correlation between polyphenol/flavonoid content and FRAP values indicates that phenolics are the main contributors to reducing activity by donating electrons and stabilizing free radicals (El‐Sayed et al. 2019; Wanyo et al. 2014). Notably, ethanolic extract was more efficient than aqueous, reflecting its broader phenolic profile. These results emphasize that rice bran extracts not only increase antioxidant content but also translate into measurable functional improvements in oxidative stability.

Overall, the consistent and concentration‐dependent improvements in TPC, TFC, DPPH, and FRAP clearly demonstrate that rice bran extracts—particularly ethanolic—are effective natural antioxidant sources. Their incorporation into protein bars not only enhances nutritional quality but also contributes to functional food development with extended shelf life and potential health‐promoting effects. Key phenolic constituents, such as ferulic acid and γ‐oryzanol, likely underpin these effects by providing strong antioxidant activity and interacting with the protein matrix, which may influence texture, flexibility, and sensory perception of the bars (Javed et al. 2025; Jun et al. 2012; Laokuldilok et al. 2011).

### 3.8 Microbial Quality

As shown in Table 6, incorporation of rice bran extracts reduced microbial load compared to the control, likely because of phenolics and flavonoids forming complexes with microbial proteins and cell walls (Asif and Khodadadi 2013). A concentration‐dependent decline was observed for both extracts, with the ethanolic extract exerting stronger effects because of its higher antimicrobial compound content and the inherent antimicrobial activity of ethanol (Karabasanavar et al. 2022). No mold or yeast growth was detected in any sample, confirming hygienic conditions and extract efficacy. Thus, the combined antimicrobial and antioxidant properties of rice bran extracts may synergistically enhance the safety and storage stability of protein bars.

**TABLE 6:** Microbial quality of protein bars supplemented with different levels of rice bran extracts.

Treatment		Total microbial count (cfu/g)	Yeast and mold
Control		400 ± 0.001^a^	0^a^
Aqueous	0.50%	130 ± 0.001^b^	0^a^
	1%	100 ± 0.001^c^	0^a^
	1.50%	70 ± 0.001^e^	0^a^
	2%	40 ± 0.001^g^	0^a^
Ethanolic	0.50%	100 ± 0.001^c^	0^a^
	1%	80 ± 0.001^d^	0^a^
	1.50%	60 ± 0.001^f^	0^a^
	2%	20 ± 0.001^h^	0^a^

*Note:* Values are presented as mean ± standard deviation (*n* = 3). Different superscript letters within the same column indicate significant differences among treatments at 5% significance level.

### 3.9 Texture Analysis

Texture is a key quality attribute of protein bars, influencing sensory perception, consumer acceptance, and overall product quality. It encompasses mechanical, structural, and tactile properties that arise from the micro‐ and macrostructure of the food matrix and is affected by ingredient interactions, processing conditions, storage, and water distribution (Chen and Opara 2013; Tamime et al. 2018). TPA was conducted to evaluate instrumental textural attributes, simulating mastication by compressing samples twice. The parameters measured included hardness, adhesiveness, cohesiveness, springiness (elasticity), gumminess, and chewiness (Peleg 2019). These measurements help link instrumental data to sensory perception, providing a comprehensive understanding of textural behavior in protein bars (Table 7).

**TABLE 7:** Texture properties of protein bars supplemented with different levels of rice bran extracts.

Treatment		Hardness (g)	Adhesiveness (mj)	Cohesiveness	Elasticity (mm)	Gumminess (g)	Chewiness (mJ)
Control		3640.00 ± 41.581^a^	0.20 ± 0.01^e^	0.36 ± 0.028^bc^	3.18 ± 0.347^b^	1110.00 ± 171.60^a^	33.51 ± 5.473^ab^
Aqueous	0.50%	1919.00 ± 55.32^e^	0.53 ± 0.152^cde^	0.40 ± 0.026^ab^	2.08 ± 0.312^c^	631.67 ± 58.943^b^	13.00 ± 3.004^c^
	1%	2154.33 ± 19.347^d^	0.63 ± 0.152^bcd^	0.378 ± 0.018^bc^	2.98 ± 0.312^bc^	710.67 ± 171.103^b^	21.30 ± 5.950^abc^
	1.50%	2417.10 ± 39.295^c^	1.03 ± 0.115^a^	0.40 ± 0.011^ab^	2.22 ± 0.332^bc^	771.67 ± 869^ab^	19.42 ± 7.562^bc^
	2%	2629.33 ± 10.692^b^	0.33 ± 0.152^de^	0.33 ± 0.032^bc^	5.00 ± 0.559^a^	786.67 ± 135.537^ab^	38.39 ± 6.548^a^
Ethanolic	0.50%	1972.77 ± 36.136^e^	0.53 ± 0.152^cde^	0.35 ± 0.034^bc^	2.16 ± 0.145^bc^	582.43 ± 133.610^b^	14.73 ± 7.093^c^
	1%	1671.10 ± 35.822^f^	0.96 ± 0.152^ab^	0.31 ± 0.035^c^	2.32 ± 0.460^bc^	795.33 ± 67.678^a^	18.56 ± 3.690^bc^
	1.50%	1744.23 ± 60.680^f^	0.30 ± 0.001^de^	0.46 ± 0.026^a^	4.46 ± 0.32 ^a^	715.90 ± 76.867^b^	18.52 ± 5.904^bc^
	2%	1512.00 ± 5.196^g^	0.83 ± 0.057^abc^	0.35 ± 0.020^bc^	2.06 ± 0.453^c^	926.33 ± 154.610^ab^	22.00 ± 7.196^abc^

*Note:* Values are presented as mean ± standard deviation (*n* = 3). Different superscript letters within the same column indicate significant differences among treatments at 5% significance level.

Hardness reflects the force required to deform the sample. Ethanolic extract‐fortified bars exhibited lower hardness compared to the control, likely due to protein–polyphenol interactions that inhibit disulfide bond formation and aggregation, along with enhanced moisture retention and plasticizing effects of polyphenols. In contrast, bars with aqueous extracts showed slightly higher hardness at increased concentrations, as starch and fiber components promoted water redistribution and network stabilization, leading to a denser structure (Alba et al. 2019; Cao and Xiong 2017).

Adhesiveness, indicating the work required to overcome surface stickiness, increased slightly in extract‐containing bars, especially with aqueous extracts. This effect can be attributed to enhanced covalent and non‐covalent bonding between proteins and polyphenols, which improves matrix cohesion and water retention (Malik et al. 2018; Tang et al. 2025).

Cohesiveness, reflecting the internal structural integrity, increased marginally in all extract‐containing bars because of strengthened protein networks through polyphenol interactions (Malik et al. 2018). Aqueous extract bars exhibited slightly higher cohesiveness, likely because of additional starch‐mediated network reinforcement.

Springiness (elasticity), the ability to recover shape after deformation, improved in most extract‐fortified samples. Polyphenolic compounds reinforced gel networks through hydrogen bonding, enhancing viscosity and flexibility. At higher polyphenol levels, competitive binding with proteins sometimes reduced elasticity, particularly in ethanolic extract bars at 2% concentration. Aqueous extract bars, enriched in starch, displayed higher springiness because of improved three‐dimensional network formation and gel stabilization (Deng et al. 2023; Han et al. 2020).

Gumminess, defined as the product of hardness and cohesiveness, generally decreased in ethanolic extract bars because of softened structures and reduced matrix resistance. Aqueous extract bars showed slightly higher gumminess, as starch filled interstitial spaces, enhancing network integrity and water retention (Malik et al. 2018; Tang et al. 2025).

Chewiness, dependent on hardness, cohesiveness, and elasticity, was highest in control and 2% aqueous extract bars. Ethanolic extract bars exhibited lower chewiness because of the softening effects of polyphenols and moisture retention, whereas aqueous extract bars demonstrated increased chewiness with higher starch content and denser networks (Ngo et al. 2022; Xue et al. 2024).

Overall, these results demonstrate that the type and concentration of rice bran extract can effectively modulate textural properties of protein bars. Ethanolic extracts favor softer, more flexible textures, whereas aqueous extracts produce firmer, cohesive structures. The observed changes are explained by the interplay of polyphenol–protein interactions, starch and fiber content, moisture migration, and network stabilization, highlighting the potential of rice bran extracts to fine‐tune both instrumental and sensory textural attributes in functional protein bars (Diaz et al. 2021; Jiang et al. 2021; Meng and Li 2021).

### 3.10 Color Measurement

Color parameters of protein bars (L*, a*, b*, ΔE, and browning index) were significantly affected by the addition of rice bran extracts (Table 8). Incorporation of both ethanolic and aqueous extracts decreased L* values (darker color) and increased a* and b* values compared to the control, indicating enhanced redness and yellowness. These changes reflect the chromogenic nature of polyphenols and flavonoids, as well as their ability to participate in oxidative and non‐enzymatic browning reactions (Peanparkdee and Iwamoto 2019).

**TABLE 8:** Color properties of protein bars supplemented with different levels of rice bran extracts.

Treatment		L*	a*	b*	ΔE	BI
Control		35.12 ± 0.051^a^	19.11 ± 0.142^h^	22.47 ± 0.014^i^	45.93 ± 0.086^i^	132.72 ± 0.442^g^
Aqueous	0.50%	34.70 ± 0.106^b^	22.56 ± 0.047^f^	23.30 ± 0.016^h^	47.49 ± 0.089^h^	143.93 ± 0.607^d^
	1%	33.90 ± 0.040^d^	24.36 ± 0.102^e^	24.20 ± 0.048^f^	48.25 ± 0.137^f^	146.80 ± 0.284^c^
	1.50%	33.06 ± 0.031^f^	26.92 ± 0.005^c^	25.30 ± 0.035^d^	49.41 ± 0.011^d^	154.51 ± 0.618^a^
	2%	32.00 ± 0.005^h^	29.75 ± 0.160^b^	26.50 ± 0.049^c^	51.97 ± 0.126^b^	155.38 ± 0.028^a^
Ethanolic	0.50%	34.50 ± 0.105^c^	21.93 ± 0.110^g^	**23.80 ± 0.040^g^	47.24 ± 0.126^g^	136.27 ± 0.745^f^
	1%	33.50 ± 0.091^e^	22.03 ± 0.084^g^	24.94 ± 0.030^e^	48.11 ± 0.106^e^	140.28 ± 0.235^e^
	1.50%	32.44 ± 0.087^g^	25.33 ± 0.014^d^	26.30 ± 0.023^b^	50.22 ± 0.080^c^	149.56 ± 0.315^b^
	2%	31.15 ± 0.066^i^	30.51 ± 0.185^a^	27.03 ± 0.041^a^	51.44 ± 0.006^a^	152.93 ± 0.123^a^

*Note:* Values are presented as mean ± standard deviation (*n* = 3). Different superscript letters within the same column indicate significant differences among treatments at 5% significance level.

Ethanolic extract caused more pronounced color shifts than aqueous extract, consistent with its higher phenolic and flavonoid content (Ghasemzadeh et al. 2015). However, the browning index was highest in the 2% aqueous extract treatment. This can be explained by the greater instability and oxidation of water‐soluble phenolics, which actively contribute to Maillard‐type browning reactions during formulation. Thus, although ethanolic extract intensified chromatic changes through its phenolic load, aqueous extract promoted stronger browning through accelerated oxidative pathways (Bork et al. 2022; Sukhonthara et al. 2016). These compositional differences demonstrate how solvent polarity and extract chemistry determine final product appearance.

### 3.11 Sensory Evaluation

Six sensory attributes (color, taste, odor, chewiness, texture, and overall acceptability) were evaluated by trained panelists (Table 9). All samples received mean scores above 4.0, indicating good acceptability across treatments. However, distinct trends emerged depending on extract type and concentration.

**TABLE 9:** Sensory properties of protein bars supplemented with different levels of rice bran extracts.

Treatment		Color	Taste	Odor	Chewiness	Texture	Overall acceptability
Control		4.46 ± 0.612^f^	4.20 ± 0.816^a^	3.46 ± 0.833^bc^	4.26 ± 0.703^a^	4.18 ± 0.457^f^	4.11 ± 0.845^c^
Aqueous	0.50%	4.51 ± 0.617^e^	4.06 ± 0.828^b^	4.00 ± 0.845^a^	4.26 ± 0.703^a^	4.53 ± 0.457^d^	4.27 ± 0.743^b^
	1%	4.60 ± 0.632^d^	3.91 ± 0.861^e^	4.06 ± 0.798^a^	4.26 ± 0.703^a^	4.53 ± 0.457^d^	4.27 ± 0.743^b^
	1.50%	4.66 ± 0.617^c^	3.60 ± 0.798^f^	3.73 ± 0.961^ab^	4.13 ± 0.639^a^	4.53 ± 0.457^d^	4.13 ± 0.617^c^
	2%	4.66 ± 0.632^b^	3.60 ± 1.014^f^	3.60 ± 0.828^bc^	4.40 ± 0.632^a^	4.50 ± 0.457^e^	4.15 ± 0.774^c^
Ethanolic	0.50%	4.53 ± 0.632^e^	4.20 ± 0.676^a^	4.00 ± 0.755^a^	4.46 ± 0.639^a^	4.60 ± 0.457^c^	4.35 ± 0.703^a^
	1%	4.66 ± 0.617^e^	4.00 ± 0.755^c^	3.73 ± 0.798^ab^	4.33 ± 0.617^a^	4.66 ± 0.455^b^	4.27 ± 0.816^b^
	1.50%	4.53 ± 0.632^d^	3.93 ± 0.798^d^	3.73 ± 0.883^ab^	4.40 ± 0.632^a^	4.66 ± 0.457^b^	4.25 ± 0.723^b^
	2%	4.73 ± 0.632^a^	3.26 ± 0.915^g^	3.26 ± 0.703^c^	4.33 ± 0.617^a^	4.68 ± 0.415^a^	4.05 ± 0.703^d^

*Note:* Values are presented as mean ± standard deviation (*n* = 3). Different superscript letters within the same column indicate significant differences among treatments at 5% significance level.

#### 3.11.1 Color

Panelists' perception of color aligned with instrumental measurements. Samples containing ethanolic extracts (particularly 2% and 1.5%) received the highest scores, suggesting that the darker hue imparted by phenolics was considered visually appealing. By contrast, aqueous extract and control samples scored lower, indicating that moderate darkening improved rather than diminished color acceptability.

#### 3.11.2 Taste

Taste scores were concentration‐dependent. The control and low‐extract treatments (0.5%) achieved the highest ratings, whereas higher concentrations—especially 2% ethanolic extract—scored lowest (3.26). This reflects the dual effect of phenolics: at low levels they fortify flavor complexity, but at high levels their inherent bitterness reduces palatability (Martillanes, Ramírez, et al. 2020).

#### 3.11.3 Odor

Odor was only moderately affected by extracts. Both aqueous and ethanolic extracts at 0.5%–1% slightly improved odor compared to the control, possibly because of volatile compounds released from rice bran. Aqueous extracts tended to score marginally higher, but overall differences were not statistically significant, keeping odor within the “moderate to good” range.

#### 3.11.4 Chewiness

Chewiness scores (4.13–4.46) showed no significant differences among treatments. This suggests that the variations in instrumental texture (hardness, elasticity) were not strong enough to alter consumer perception of chewability, and all samples were rated “good to very good.”

#### 3.11.5 Texture

Texture was one of the most positively influenced attributes. The control scored lowest (4.18), whereas bars containing ethanolic extract—especially at 2%—achieved the highest scores (4.69). Panelists perceived extract fortification as improving softness and structural uniformity. Mechanistically, polyphenol–protein interactions enhance solubility and emulsifying capacity, generating stable, elastic networks (Meng and Li 2021). In contrast, the higher carbohydrate content of aqueous extracts slightly reduced scores, likely because of firmer, less elastic textures arising from competition for water absorption.

#### 3.11.6 Overall Acceptability

Overall acceptability scores confirmed the above trends. All treatments were well accepted (≥ 4.0), but the 1% ethanolic extract sample ranked highest (4.35), balancing enhanced color and texture with acceptable taste. Conversely, the 2% aqueous extract scored lowest (4.05), reflecting lower flavor and odor desirability despite acceptable texture. These findings suggest that moderate inclusion of ethanolic rice bran extract achieves the best compromise between functionality and consumer appeal.

### 3.12 Secondary Structures

Protein secondary structures—including α‐helix, intramolecular and intermolecular β‐sheets, β‐turns, and random coils—are directly linked to the functional behavior of food proteins. Alterations in these conformations affect molecular packing, water–protein interactions, and the strength of the three‐dimensional protein matrix, thereby influencing textural attributes such as hardness, cohesiveness, elasticity, and chewiness, as well as sensory perception (Movaghar, Pourfarzad, Nikoo, 2025; Pourghasemian et al. 2025a; Talemi et al. 2025). Therefore, FTIR‐derived structural changes provide mechanistic insight into how rice bran extracts modulate the physicochemical behavior of fortified protein bars (Table 10).

**TABLE 10:** Secondary structure of protein bars supplemented with different levels of rice bran extracts.

Treatment		α‐Helices (%)	Intramolecular β‐sheets (%)	Intermolecular β‐Sheets (%)	β‐Turns (%)	Random coils (%)
Control		39.62 ± 0.120^a^	27.75 ± 0.080^f^	6.71 ± 0.090^a^	16.31 ± 0.090^e^	9.61 ± 0.050^d^
Aqueous	0.50%	38.19 ± 0.110^b^	28.24 ± 0.090^e^	6.28 ± 0.050^b^	17.08 ± 0.080^d^	10.21 ± 0.060^c^
	1%	38.76 ± 0.100^b^	28.11 ± 0.080^e^	**6.22 ± 0.060^b^	16.87 ± 0.090^e^	10.04 ± 0.060^c^
	1.50%	39.22 ± 0.100^a^	27.98 ± 0.070^e^	6.32 ± 0.060^a^	16.55 ± 0.090^e^	9.93 ± 0.050^c^
	2%	39.46 ± 0.090^a^	27.84 ± 0.060^f^	6.51 ± 0.060^a^	16.42 ± 0.080^e^	9.76 ± 0.050^d^
Ethanolic	0.50%	36.57 ± 0.100^c^	28.56 ± 0.080^d^	6.01 ± 0.050^b^	18.04 ± 0.090^c^	10.82 ± 0.060^b^
	1%	34.77 ± 0.100^d^	29.07 ± 0.090^c^	**5.59 ± 0.040^c^	19.18 ± 0.080^b^	11.39 ± 0.060^b^
	1.50%	33.03 ± 0.100^e^	29.74 ± 0.080^b^	5.09 ± 0.050^d^	20.16 ± 0.090^a^	11.98 ± 0.060^a^
	2%	31.00 ± 0.090^f^	30.40 ± 0.090^a^	4.80 ± 0.080^e^	21.20 ± 0.080^a^	12.60 ± 0.070^a^

*Note:* Values are presented as mean ± standard deviation (*n* = 3). Different superscript letters within the same column indicate significant differences among treatments at 5% significance level.

#### 3.12.1 α‐Helices

The α‐helix represents an ordered, compact conformation that supports rigidity and cohesive network formation. Samples with higher α‐helix content exhibited firmer textures, consistent with the control sample. Aqueous extract treatments slightly increased α‐helix proportions, suggesting stabilization of ordered domains through fiber‐ and polysaccharide‐mediated hydrogen bonding. This stabilization likely enhanced structural rigidity, aligning with the slightly higher hardness and chewiness of aqueous extract bars. In contrast, ethanolic extracts substantially reduced α‐helix content, indicating partial unfolding of helical segments. Phenolic compounds can disrupt intrachain hydrogen bonds and interfere with disulfide bond formation, reducing matrix compactness. This explains the softer, less cohesive texture and higher elasticity of bars containing ethanolic extracts (Beh et al. 2021; Muley and Singhal 2020). The FTIR results thus closely match the instrumental TPA findings.

#### 3.12.2 Intramolecular β‐Sheets

Intramolecular β‐sheets contribute to internal stability within individual protein molecules. Ethanolic extract treatments increased this structural element, suggesting that phenolic–protein interactions induced a reorganization of backbone hydrogen bonding. Although this increase appeared stabilizing, the resulting structure tended to be flexible rather than firm, because enhanced intrachain stabilization occurred simultaneously with reduced interchain connectivity. This duality explains why ethanolic samples were softer yet resilient and elastic: the proteins became internally stable but less aggregated, enabling greater deformation and recovery during chewing (Gao et al. 2024).

#### 3.12.3 Intermolecular β‐Sheets

Intermolecular β‐sheets represent protein–protein crosslinks critical for matrix rigidity, hardness, and gumminess. Aqueous extracts promoted slight increases in intermolecular β‐sheets, likely because of the combined effects of starch and fibers bridging protein chains and encouraging ordered aggregation. This corresponds well with their firmer, more cohesive textural profile. Conversely, ethanolic extracts significantly reduced intermolecular β‐sheet formation. Phenolic compounds can compete with proteins for hydrophobic and hydrogen‐bonding sites, reducing protein–protein association and resulting in a weaker, more open network (Gao et al. 2024). This structural weakening explains the lower hardness, reduced gumminess, and softer mouthfeel of ethanolic extract bars. Thus, this FTIR signature directly mirrors the TPA trends and sensory perception of softness in these formulations.

#### 3.12.4 β‐Turns

β‐turns introduce localized flexibility, enabling proteins to bend or reorient within the matrix. Ethanolic extract treatments produced a marked increase in β‐turns, reflecting greater conformational looseness caused by helix disruption and reduced crosslinking. This conformational flexibility is consistent with the observed increase in springiness and decreased chewiness, as a more flexible matrix deforms and rebounds more readily. Aqueous extracts showed minor reductions in β‐turns, consistent with their more ordered and compact structure (Liu et al. 2019; Shahidi and Dissanayaka 2023).

#### 3.12.5 Random Coils

Random coils represent disordered, unfolded regions commonly associated with reduced structural integrity. Aqueous extracts slightly decreased random coil content, supporting the interpretation that they enhanced structural order through starch‐mediated network strengthening. This structural ordering aligns with their higher hardness and chewiness. Ethanolic extracts increased random coil proportions, indicating disruption of ordered domains and formation of a more amorphous matrix. This structural disorder corresponds directly with softer textures, lower hardness, and a smoother mouthfeel (Alston et al. 2023; Li et al. 2024).

Together, the FTIR and texture results reveal a coherent mechanistic trend, indicating that an increase in random coils and β‐turns is associated with greater flexibility and softness, whereas higher levels of α‐helix and intermolecular β‐sheets correspond to increased firmness and cohesiveness.

### 3.13 Optimization

A multiple‐response optimization was performed to determine the optimal levels of rice bran extract and its type (aqueous or ethanolic) for achieving the desired quality attributes of protein bars. In this optimization process, various physicochemical, structural, antioxidant, microbial, and sensory parameters were considered simultaneously. Each response was assigned a specific goal, target range, and relative importance on the basis of its influence on product quality and consumer acceptance.

The optimization aimed to maximize key desirable attributes, including sensory properties (overall acceptability, color, texture, taste, odor, and chewiness), instrumental textural parameters (cohesiveness, springiness, and gumminess), antioxidant activities (DPPH and FRAP), bioactive compound levels (TPC and TFC), protein content, L* value, and beneficial protein secondary structures (β‐turn, intramolecular β‐sheet, and random coil). Conversely, minimization targets were set for undesirable attributes such as hardness, adhesiveness, chewiness (instrumental), browning index, acidity, peroxide value, water activity, ΔE, yeast count, and other microbial parameters, which could negatively affect texture, stability, or shelf life. Additional constraints were applied to ensure product stability, including acceptable ranges for color parameters, moisture, and minor texture indicators.

The optimization was conducted using a weighted desirability approach, where each parameter contributed to the overall desirability score according to its assigned importance and weight. Responses with higher consumer relevance or functional significance (e.g., overall acceptability, texture, and antioxidant activity) were given greater weight to guide the optimization toward a balanced formulation.

The optimization results indicated that the sample containing 1% ethanolic rice bran extract, followed by the sample containing 1% aqueous extract, exhibited the best overall performance in terms of improving quality attributes, sensory acceptability, and product stability. Validation experiments confirmed that the predicted and observed values for all measured responses were in close agreement (*p* < 0.05), demonstrating the reliability of the optimization model (data were not shown).

This multi‐response optimization highlights the critical role of extract type and concentration in simultaneously improving sensory quality, physicochemical properties, antioxidant activity, and protein structural integrity, providing a rational basis for designing protein bars with enhanced functionality and consumer appeal.

### 3.14 Principal Component Analysis

**FIGURE 1:**
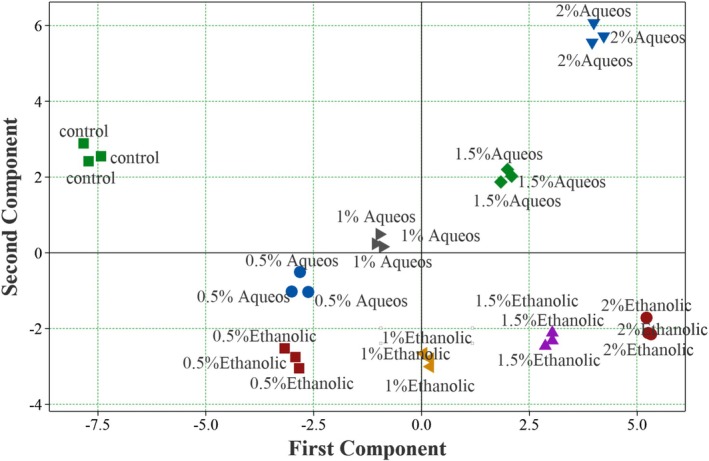
Score plot of principal component analysis on properties of protein bars containing aqueous and ethanolic rice bran extracts.

To better elucidate the interrelationships among the measured variables, the physicochemical, antioxidant, microbial, colorimetric, and sensory attributes of the protein bars were subjected to PCA (Figure 1). The analysis reduced the dataset into five meaningful principal components, with the first three components (PC1–PC3) together explaining 81.2% of the total variance. This indicates that the majority of variability among the samples could be captured within three dimensions, sufficient for interpreting the main quality differences.

PC1 (41.7% of variance) was positively associated with antioxidant and bioactive indicators, including TPC, TFC, FRAP, and color parameters (L*, a*, b*, and ΔE), while showing negative loadings for moisture, water activity, acidity, and peroxide value. This component mainly reflected a trade‐off between enhanced functional/antioxidant quality and reduced susceptibility to oxidative and microbial deterioration.

PC2 (22.0% of variance) was more closely related to mechanical and sensory characteristics. Positive loadings were observed for hardness, chewiness, cohesiveness, and ash, whereas taste, acceptability, and certain protein secondary structures (β‐turns, intra β‐sheets, and random coils) loaded negatively. This suggests that PC2 describes the contrast between stronger, more rigid textures and consumer‐preferred sensory attributes.

PC3 (17.5% of variance) separated variables associated with gumminess, chewiness, yeast count, and protein content from those linked with sensory quality (odor and taste) and antioxidant capacity (DPPH). This dimension highlighted the influence of protein fortification and microbial growth versus perceived flavor and radical scavenging efficiency.

**FIGURE 2:**
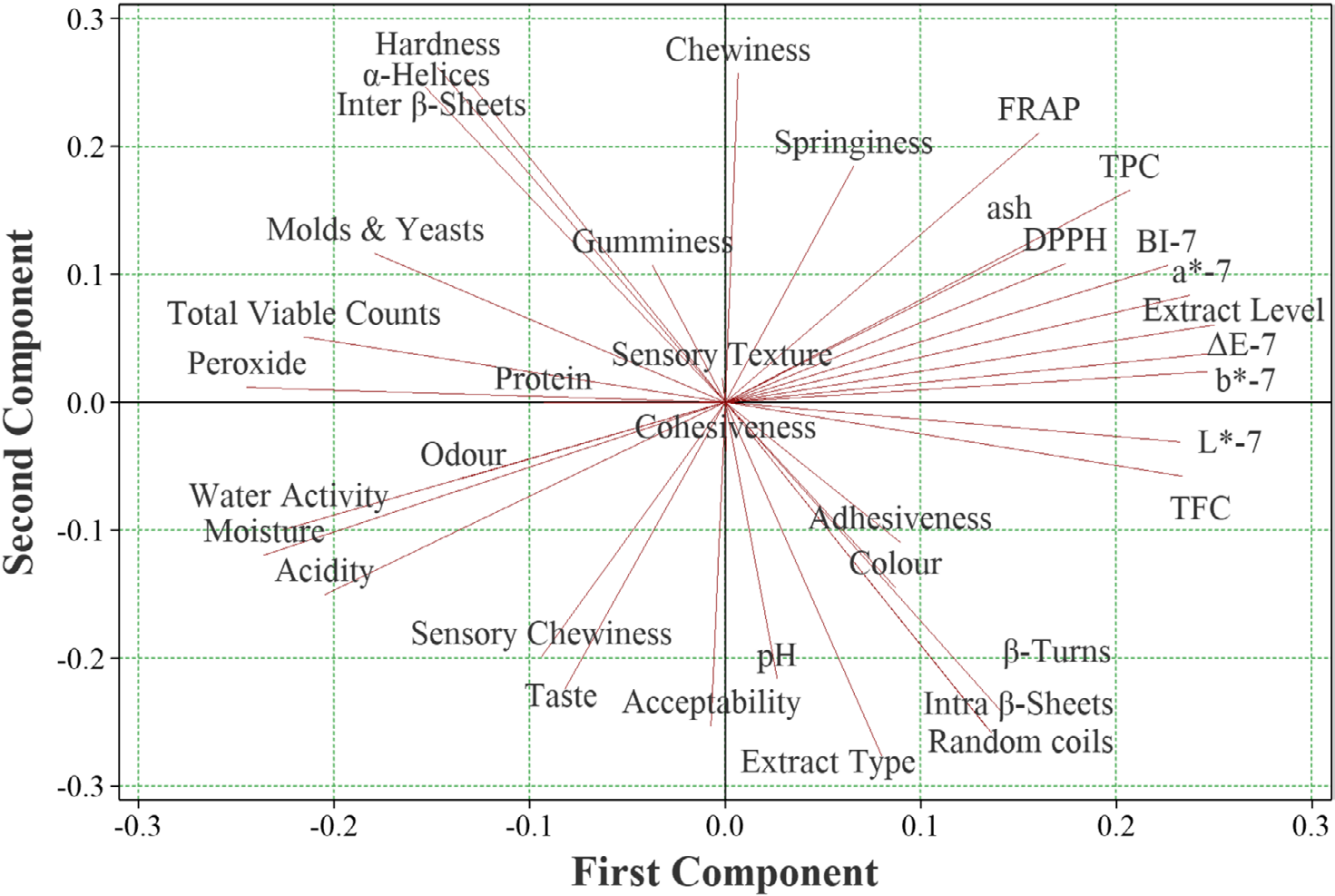
Biplot of principal component analysis on properties of protein bars containing aqueous and ethanolic rice bran extracts.

The biplot (Figure 2) clearly distinguished the extract‐fortified samples from the control. Bars containing 1% ethanolic extract were located in the quadrant associated with high antioxidant capacity and sensory acceptance, reflecting their dual contribution to functionality and consumer‐oriented quality. Samples with 1% aqueous extract also clustered positively but to a lesser extent in terms of antioxidant strength. In contrast, the control group without extract loaded negatively on PC1, indicating limited bioactivity and greater susceptibility to oxidative changes.

Overall, PCA findings were in strong agreement with the optimization outcomes. Both multivariate and response surface analyses confirmed that ethanolic and, to a lesser degree, aqueous rice bran extracts improved the functional (TPC, TFC, FRAP, and DPPH), oxidative (lower peroxide and acidity), and sensory (odor, taste, and acceptability) attributes while maintaining acceptable texture and color. These results underscore the role of rice bran extracts in simultaneously enhancing product stability, functionality, and consumer appeal, thus supporting their practical application in protein bar formulations.

### 3.15 Correlation of FTIR‐Analyzed Secondary Structures With Physicochemical, Microbial, and Sensory Properties Using the PLSR Method

Multivariate statistical approaches are powerful tools for elucidating the associations between FTIR‐analyzed protein secondary structures and the physicochemical, microbial, and sensory attributes of brown rice protein bars. These approaches are broadly classified into asymmetric and symmetric methods. Asymmetric methods, such as Partial Least Squares Regression (PLSR), are particularly valuable for predicting one dataset on the basis of another, whereas symmetric methods such as PCA are more suited for describing overall data relationships without predictive modeling (Mahesh et al. 2014).

Several studies have applied PCA and PLSR to correlate chemical, rheological, and sensory characteristics with the quality and shelf life of food products (Pourfarzad and Kisomi 2020; Pourfarzad and Taleb Derakhshan 2021; Pourfarzad and Yousefi 2021). In this study, PLSR was employed to establish predictive relationships between secondary structure elements (X) and physicochemical, microbial, and sensory responses (Y). Such correlations are highly valuable in the food industry, as protein structural characteristics fundamentally influence product quality, consumer acceptability, and stability (da Barroso Silva et al. 2020).

On the basis of their strong correlations with secondary structures, seven representative response variables were selected for modeling: hardness, browning index (BI), peroxide value, total polyphenols, overall acceptability, total energy, and total viable count.

To ensure model reliability, all PLSR models were cross‐validated using leave‐one‐out cross‐validation (LOOCV). In this approach, each sample was sequentially excluded from the calibration set, the model was refitted, and predictions were generated for the omitted sample. The resulting Q^2^ values and residuals confirmed that the models were robust, not overfitted, and suitable for predicting quality parameters from FTIR data. The relatively high coefficients of determination (*R* ^2^ values) further support the predictive power of these models. The predictive regression models are as follows:

(6) $$\begin{array}{cc} \text{Hardnes}\; & =218102-6754\;\text{Random coils}-236\;\beta -\text{Turns}-988\\ & \;\text{Inter}\;\beta -\text{Sheets}-1457\;\text{Intra}\;\beta -\text{Sheets}2498\;\alpha -\text{Helices};\\ & \left(R^{2}=82.12\%\right) \end{array}$$

(7) $$\begin{array}{cc} \mathrm{BI}=\; & -1179+4.9\;\text{Random coils}-7.7\;\beta -\text{Turns}-34.9\\ & \;\text{Inter}\;\beta -\text{Sheets}+37.3\;\text{Intra}\;\beta -\text{Sheets}+14.9\;\alpha -\text{Helices};\\ & \left(R^{2}=73.12\%\right) \end{array}$$

(8) $$\begin{array}{cc} \text{Peroxide Value}\; & =14+1.60\;\text{Random coils}-0.98\;\beta -\text{Turns}\\ & +2.77\;\text{Inter}\;\beta -\text{Sheets}-0.30\;\text{Intra}\;\beta -\text{Sheets}-0.54\;\alpha \\ & -\text{Helices};\left(R^{2}=76.27\%\right) \end{array}$$

(9) $$\begin{array}{cc} \text{Total Polyphenols}\; & =-13.1-0.004\;\text{Random coils}-0.010\;\beta -\text{Turns}\\ & -0.336\;\text{Inter}\;\beta -\text{Sheets}+0.337\;\text{Intra}\;\beta -\text{Sheets}\\ & +0.161\;\alpha -\text{Helices};\left(R^{2}=81.10\%\right) \end{array}$$

(10) $$\begin{array}{cc} \text{Total viable count}\; & =48230-1230\;\text{Random coils}-36\;\beta \\ & -\text{Turns}+94\;\text{Inter}\;\beta -\text{Sheets}-471\;\text{Intra}\;\beta \\ & -\text{Sheets}-582\;\alpha -\text{Helices};\left(R^{2}=83.22\%\right) \end{array}$$

(11) $$\begin{array}{cc} \text{Total Acceptability}\; & =-78.3+2.482\;\text{Random coils}+0.104\;\beta \\ & -\text{Turns}+0.219\;\text{Inter}\;\beta -\text{Sheets}+0.557\;\text{Intra}\;\beta \\ & -\text{Sheets}+1.003\;\alpha -\text{Helices};\left(R^{2}=87.19\%\right) \end{array}$$

(12) $$\begin{array}{cc} \text{Total energy}\; & =237+16.0\;\text{Random coils}-9.83\;\beta \\ & -\text{Turns}+18.33\;\text{Inter}\;\beta -\text{Sheets}\\ & +6.10\;\text{Intra}\;\beta -\text{Sheets}-1.79\;\alpha \\ & -\text{Helices};\left(R^{2}=75.21\%\right) \end{array}$$

These findings highlight that FTIR‐derived secondary structure data can reliably predict key physicochemical, microbial, and sensory parameters, providing a valuable approach for monitoring and optimizing the quality of protein‐based functional foods.

## 4 Conclusion

The present study highlighted the potential of aqueous and ethanolic rice bran extracts as functional ingredients in protein bars formulated with brown rice flour. Incorporation of these extracts improved overall quality, stability, and consumer‐oriented properties of the product. Bars enriched with ethanolic extract maintained higher moisture during storage and showed stronger microbial inhibition, which can be attributed to the antimicrobial and antioxidant actions of polyphenolic compounds.

From a textural perspective, extract supplementation—particularly with ethanolic extract—led to softer, more elastic bars with enhanced chewability. Color measurements and browning index confirmed that both extract types influenced visual attributes, with ethanolic extract causing more distinct modifications. Sensory evaluation further supported these findings, as bars with 1% ethanolic extract achieved the highest scores in flavor, aroma, texture, and overall acceptability.

Structural analysis via FTIR demonstrated that extracts induced rearrangements in protein secondary structures, notably in α‐helices, β‐sheets, and random coils. Although ethanolic extract promoted a more flexible protein network, the aqueous extract favored the preservation of a denser structural conformation.

Taken together, the results indicate that rice bran extracts—especially ethanolic extract—hold considerable promise as multifunctional additives and natural preservatives in protein‐rich foods. Beyond enhancing nutritional and sensory quality, their use also contributes to the valorization of rice bran as an underutilized agro‐industrial by‐product, offering both health and economic benefits.

## Author Contributions

All the authors contributed in a similar way in all steps of the manuscript, that is, conceptualization, data curation, formal analysis, funding acquisition, investigation, methodology, project administration, resources, software, supervision, validation, visualization, writing – original draft, and writing – review and editing.

## Funding

This study was financially supported by the Guilan Science and Technology Park [grant number 1404/236].

## Ethics Statement

The study involving human participants (sensory panelists) was conducted in accordance with institutional ethical standards, and informed consent was obtained from all individual participants included in the study.

## Consent

All human participants involved in the sensory evaluation study provided informed consent prior to their participation.

## Conflicts of Interest

The authors declare no conflicts of interest.

## Data Availability

The data that support the findings of this study are available from the corresponding author upon reasonable request.

## References

[fsn371537-bib-0001] Abdel‐Salam, F. F. , R. M. Ibrahim , and M. Ali . 2022. “Formulation and Evaluation of High Energy‐Protein Bars as a Nutritional Supplement for Sports Athletics.” American Journal of Food Technology 10: 53–65.

[fsn371537-bib-0002] Adámek, M. , A. Adámková , J. Mlček , M. Borkovcová , and M. Bednářová . 2018. “Acceptability and Sensory Evaluation of Energy Bars and Protein Bars Enriched With Edible Insect.” Potravinarstvo Slovak Journal of Food Sciences 12, no. 1: 431–437.

[fsn371537-bib-0003] Adrar, N. S. , K. Madani , and S. Adrar . 2019. “Impact of the Inhibition of Proteins Activities and the Chemical Aspect of Polyphenols‐Proteins Interactions.” PharmaNutrition 7: 100142.

[fsn371537-bib-0004] Alba, K. , G. M. Campbell , and V. Kontogiorgos . 2019. “Dietary Fibre From Berry‐Processing Waste and Its Impact on Bread Structure: A Review.” Journal of the Science of Food and Agriculture 99, no. 9: 4189–4199. 30737794 10.1002/jsfa.9633

[fsn371537-bib-0005] Alston, J. J. , G. M. Ginell , A. Soranno , and A. S. Holehouse . 2023. “The Analytical Flory Random Coil Is a Simple‐To‐Use Reference Model for Unfolded and Disordered Proteins.” Journal of Physical Chemistry B 127, no. 21: 4746–4760. 37200094 10.1021/acs.jpcb.3c01619 PMC10875986

[fsn371537-bib-0006] Andriani, R. , T. Subroto , S. Ishmayana , and D. Kurnia . 2022. “Enhancement Methods of Antioxidant Capacity in Rice Bran: A Review.” Food 11, no. 19: 2994. 10.3390/foods11192994 PMC9564381 36230070

[fsn371537-bib-0007] Antunes, L. L. , A. L. Back , M. L. B. C. Kossar , A. G. Spessato , E. Colla , and D. A. Drunkler . 2023. “Prebiotic Potential of Carbohydrates From Defatted Rice Bran–Effect of Physical Extraction Methods.” Food Chemistry 404: 134539. 36242965 10.1016/j.foodchem.2022.134539

[fsn371537-bib-0008] Asif, M. , and E. Khodadadi . 2013. “Medicinal Uses and Chemistry of Flavonoid Contents of Some Common Edible Tropical Plants.” Archives of Advances in Biosciences 4, no. 3: 119–138.

[fsn371537-bib-0009] Baliyan, S. , R. Mukherjee , A. Priyadarshini , et al. 2022. “Determination of Antioxidants by DPPH Radical Scavenging Activity and Quantitative Phytochemical Analysis of *Ficus religiosa* .” Molecules 27, no. 4: 1326. 35209118 10.3390/molecules27041326 PMC8878429

[fsn371537-bib-0010] Beh, C. , E. Cheng , N. M. Nasir , et al. 2021. “Dielectric and Material Analysis on Physicochemical Activity of Porous Hydroxyapatite/Cornstarch Composites.” International Journal of Biological Macromolecules 166: 1543–1553. 33181217 10.1016/j.ijbiomac.2020.11.034

[fsn371537-bib-0107] Bhat, F. M. , and C. S. Riar . 2017. “Extraction, identification and assessment of antioxidative compounds of bran extracts of traditional rice cultivars: An analytical approach.” Food Chemistry 237: 264–274. 10.1016/j.foodchem.2017.05.113. 28763995

[fsn371537-bib-0011] Bhanger, M. I. , S. Iqbal , F. Anwar , M. Imran , M. Akhtar , and M. Zia‐ul‐Haq . 2008. “Antioxidant Potential of Rice Bran Extracts and Its Effects on Stabilisation of Cookies Under Ambient Storage.” International Journal of Food Science & Technology 43, no. 5: 779–786.

[fsn371537-bib-0012] Bhosale, S. , and D. Vijayalakshmi . 2015. “Processing and Nutritional Composition of Rice Bran.” Current Research in Nutrition and Food Science Journal 3, no. 1: 74–80.

[fsn371537-bib-0013] Bork, L. V. , S. Rohn , and C. Kanzler . 2022. “Characterization of Colorants Formed by Non‐Enzymatic Browning Reactions of Hydroxycinnamic Acid Derivatives.” Molecules 27, no. 21: 7564. 36364391 10.3390/molecules27217564 PMC9658777

[fsn371537-bib-0014] Burke, L. M. , J. A. Hawley , S. H. Wong , and A. E. Jeukendrup . 2013. “Carbohydrates for Training and Competition.” Food, Nutrition and Sports Performance III 29: 17–27. 10.1080/02640414.2011.585473 21660838

[fsn371537-bib-0015] Cao, Y. , and Y. L. Xiong . 2017. “Interaction of Whey Proteins With Phenolic Derivatives Under Neutral and Acidic pH Conditions.” Journal of Food Science 82, no. 2: 409–419. 28071787 10.1111/1750-3841.13607

[fsn371537-bib-0016] Chen, L. , and U. L. Opara . 2013. “Texture Measurement Approaches in Fresh and Processed Foods—A Review.” Food Research International 51, no. 2: 823–835.

[fsn371537-bib-0017] da Barroso Silva, F. L. , P. Carloni , D. Cheung , et al. 2020. “Understanding and Controlling Food Protein Structure and Function in Foods: Perspectives From Experiments and Computer Simulations.” Annual Review of Food Science and Technology 11: 365–387. 10.1146/annurev-food-032519-051640 PMC7379328 31951485

[fsn371537-bib-0018] Das, A. B. , V. Goud , and C. Das . 2018. “Extraction and Characterization of Phenolic Content From Purple and Black Rice (*Oryza sativa* L) Bran and Its Antioxidant Activity.” Journal of Food Measurement and Characterization 12: 332–345.

[fsn371537-bib-0019] Deng, N. , X. Bian , S. Luo , C. Liu , and X. Hu . 2023. “The Starch‐Polyphenol Inclusion Complex: Preparation, Characterization and Digestion.” Food Bioscience 53: 102655.

[fsn371537-bib-0020] Diaz, J. T. , E. A. Foegeding , and M. A. Lila . 2021. “Whey Protein‐Polyphenol Aggregate Particles Mitigate Bar Hardening Reactions in High Protein Bars.” LWT 138: 110747.

[fsn371537-bib-0021] El‐Sayed, M. , S. Awad , and A. Ibrahim . 2019. “Impact of Purslane (*Portulaca oleracea* L.) Extract as Antioxidant and Antimicrobial Agent on Overall Quality and Shelf Life of Greek‐Style Yoghurt.” Egyptian Journal of Food Science 47, no. 1: 51–64.

[fsn371537-bib-0022] Eng, H. Y. , and N. H. Mohd Rozalli . 2022. “Rice Bran and Its Constituents: Introduction and Potential Food Uses.” International Journal of Food Science & Technology 57, no. 7: 4041–4051.

[fsn371537-bib-0023] Gao, Y. , R. Liu , and H. Liang . 2024. “Food Hydrocolloids: Structure, Properties, and Applications.” Food 13: 1077. 10.3390/foods13071077 PMC11011930 38611381

[fsn371537-bib-0024] Ghasemzadeh, A. , A. Baghdadi , H. ZE Jaafar , M. K. Swamy , and P. E. Megat Wahab . 2018. “Optimization of Flavonoid Extraction From Red and Brown Rice Bran and Evaluation of the Antioxidant Properties.” Molecules 23, no. 8: 1863. 30049990 10.3390/molecules23081863 PMC6222751

[fsn371537-bib-0025] Ghasemzadeh, A. , H. Z. Jaafar , A. S. Juraimi , and A. Tayebi‐Meigooni . 2015. “Comparative Evaluation of Different Extraction Techniques and Solvents for the Assay of Phytochemicals and Antioxidant Activity of Hashemi Rice Bran.” Molecules 20, no. 6: 10822–10838. 26111171 10.3390/molecules200610822 PMC6272729

[fsn371537-bib-0026] Gul, K. , B. Yousuf , A. Singh , P. Singh , and A. A. Wani . 2015. “Rice Bran: Nutritional Values and Its Emerging Potential for Development of Functional Food—A Review.” Bioactive Carbohydrates and Dietary Fibre 6, no. 1: 24–30.

[fsn371537-bib-0027] Han, X. , M. Zhang , R. Zhang , et al. 2020. “Physicochemical Interactions Between Rice Starch and Different Polyphenols and Structural Characterization of Their Complexes.” LWT 125: 109227.

[fsn371537-bib-0028] Harris, G. K. , and M. R. Marshall . 2017. “Ash Analysis.” In Food Analysis, 287–297. Springer.

[fsn371537-bib-0029] Harwood, W. S. , and M. Drake . 2019. “Understanding Implicit and Explicit Consumer Desires for Protein Bars, Powders, and Beverages.” Journal of Sensory Studies 34, no. 3: e12493.

[fsn371537-bib-0030] Hassan, S. K. 2020. “Quantitative and Qualitative Effects of Proteins and Natural Sugars on Hardening and Color of High‐Protein Nutrition Bars During Storage.” EurAsian Journal of BioSciences 14, no. 1: 915.

[fsn371537-bib-0031] Hatami, S. , N. Tajabadi , R. Massoud , and A. Sharifan . 2023. “Chemical and Sensorial Properties of Probiotic Beverage Based on Rice Bran Extract and Honey.” Biomass Conversion and Biorefinery 13, no. 6: 5151–5156.

[fsn371537-bib-0032] Huang, W. , B. Liu , D. Shi , et al. 2024. “Research Progress on the Quality, Extraction Technology, Food Application, and Physiological Function of Rice Bran Oil.” Food 13, no. 20: 3262. 10.3390/foods13203262 PMC11507353 39456324

[fsn371537-bib-0033] Irakli, M. , F. Kleisiaris , K. Kadoglidou , and D. Katsantonis . 2018. “Optimizing Extraction Conditions of Free and Bound Phenolic Compounds From Rice By‐Products and Their Antioxidant Effects.” Food 7, no. 6: 93. 10.3390/foods7060093 PMC6024898 29899303

[fsn371537-bib-0034] Ishak, H. , H. Yoshida , N. A. Muda , M. H. S. Ismail , and S. Izhar . 2019. “Rapid Processing of Abandoned Oil Palm Trunks Into Sugars and Organic Acids by Sub‐Critical Water.” PRO 7, no. 9: 593.

[fsn371537-bib-0035] Jabeen, S. , N. Huma , A. Sameen , and M. A. Zia . 2020. “Formulation and Characterization of Protein‐Energy Bars Prepared by Using Dates, Apricots, Cheese and Whey Protein Isolate.” Food Science and Technology 41: 197–207.

[fsn371537-bib-0036] Jäger, R. , C. M. Kerksick , B. I. Campbell , et al. 2017. “International Society of Sports Nutrition Position Stand: Protein and Exercise.” Journal of the International Society of Sports Nutrition 14, no. 1: 20. 28642676 10.1186/s12970-017-0177-8 PMC5477153

[fsn371537-bib-0037] Javed, M. , J. Jawid , S. Zafar , et al. 2025. “Black Rice as the Emerging Functional Food: Bioactive Compounds, Therapeutic Potential and Industrial Applications.” Frontiers in Nutrition 12: 1705983. 41235311 10.3389/fnut.2025.1705983 PMC12605397

[fsn371537-bib-0038] Jiang, Z. , K. Wang , X. Zhao , et al. 2021. “High‐Protein Nutrition Bars: Hardening Mechanisms and Anti‐Hardening Methods During Storage.” Food Control 127: 108127.

[fsn371537-bib-0039] Jin, Y. , D. Hu , Q. Chen , et al. 2023. “Water‐Based Green and Sustainable Extraction Protocols for Value‐Added Compounds From Natural Resources.” Current Opinion in Green and Sustainable Chemistry 40: 100757.

[fsn371537-bib-0040] Jun, H. I. , G. S. Song , E. I. Yang , Y. Youn , and Y. S. Kim . 2012. “Antioxidant Activities and Phenolic Compounds of Pigmented Rice Bran Extracts.” Journal of Food Science 77, no. 7: 759–764. 10.1111/j.1750-3841.2012.02763.x 22708681

[fsn371537-bib-0041] Karabasanavar, N. , M. Chakkodabail Benakabhat , S. Agalagandi Gopalakrishna , et al. 2022. “Polyclonal Hen Egg Yolk Antibodies Could Confer Passive Protection Against Salmonella Serotypes in Broiler Chicks.” Journal of Food Safety 42, no. 4: e12987.

[fsn371537-bib-0042] Karastogianni, S. , S. Girousi , and S. Sotiropoulos . 2016. “pH: Principles and Measurement.” Encyclopedia of Food and Health 4: 333–338.

[fsn371537-bib-0043] Kaur, S. , J. Chen , and A. Ubeyitogullari . 2024. “Formation of Nanoporous Aerogels From Defatted Rice Bran via Supercritical Carbon Dioxide Drying.” Sustainable Food Technology 2, no. 1: 152–161. 10.1016/j.foodchem.2024.139833 38833864

[fsn371537-bib-0044] Laokuldilok, T. , C. F. Shoemaker , S. Jongkaewwattana , and V. Tulyathan . 2011. “Antioxidants and Antioxidant Activity of Several Pigmented Rice Brans.” Journal of Agricultural and Food Chemistry 59, no. 1: 193–199. 21141962 10.1021/jf103649q

[fsn371537-bib-0045] Lee, J.‐S. , N. Sreenivasulu , R. S. Hamilton , and A. Kohli . 2019. “Brown Rice, a Diet Rich in Health Promoting Properties.” Journal of Nutritional Science and Vitaminology 65: 26–28. 10.3177/jnsv.65.S26 31619639

[fsn371537-bib-0046] Li, H. , M. D. Tuttle , K. W. Zilm , and V. S. Batista . 2024. “Rapid Quantification of Protein Secondary Structure Composition From a Single Unassigned 1D 13C Nuclear Magnetic Resonance Spectrum.” Journal of the American Chemical Society 146, no. 40: 27542–27554. 39322561 10.1021/jacs.4c08300 PMC13300729

[fsn371537-bib-0047] Liu, J. , H. Yong , X. Yao , H. Hu , D. Yun , and L. Xiao . 2019. “Recent Advances in Phenolic–Protein Conjugates: Synthesis, Characterization, Biological Activities and Potential Applications.” RSC Advances 9, no. 61: 35825–35840. 35528080 10.1039/c9ra07808h PMC9074773

[fsn371537-bib-0048] Lu, N. , L. Zhang , X. Zhang , J. Li , T. P. Labuza , and P. Zhou . 2016. “Molecular Migration in High‐Protein Intermediate‐Moisture Foods During the Early Stage of Storage: Variations Between Dairy and Soy Proteins and Effects on Texture.” Food Research International 82: 34–43.

[fsn371537-bib-0049] Mahesh, S. , D. Jayas , J. Paliwal , and N. White . 2014. “Comparison of Partial Least Squares Regression (PLSR) and Principal Components Regression (PCR) Methods for Protein and Hardness Predictions Using the Near‐Infrared (NIR) Hyperspectral Images of Bulk Samples of Canadian Wheat.” Food and Bioprocess Technology 8: 31–40.

[fsn371537-bib-0050] Małecki, J. , K. Terpiłowski , M. Nastaj , and B. G. Sołowiej . 2022. “Physicochemical, Nutritional, Microstructural, Surface and Sensory Properties of a Model High‐Protein Bars Intended for Athletes Depending on the Type of Protein and Syrup Used.” International Journal of Environmental Research and Public Health 19, no. 7: 3923. 35409605 10.3390/ijerph19073923 PMC8997551

[fsn371537-bib-0051] Małecki, J. , I. Tomasevic , I. Djekic , and B. G. Sołowiej . 2020. “The Effect of Protein Source on the Physicochemical, Nutritional Properties and Microstructure of High‐Protein Bars Intended for Physically Active People.” Food 9, no. 10: 1467. 10.3390/foods9101467 PMC7602487 33076297

[fsn371537-bib-0052] Malik, N. , O. Gouseti , and S. Bakalis . 2018. “Effect of Freezing on Microstructure and Reconstitution of Freeze‐Dried High Solid Hydrocolloid‐Based Systems.” Food Hydrocolloids 83: 473–484.

[fsn371537-bib-0053] Mandal, S. , M. L. Rahman , P. Das , et al. 2023. “Effect of Maceration, Ultrasound, and Microwave‐Assisted Method of Extraction on Antioxidant Activity and Phenolic Profile of Free, Esterified, and Bound Phenolics of Tulaipanji Rice.” International Journal of Food Engineering 19, no. 12: 631–640.

[fsn371537-bib-0054] Martillanes, S. , R. Ramírez , G. Amaro‐Blanco , M. C. Ayuso‐Yuste , M. V. Gil , and J. Delgado‐Adámez . 2020. “Effect of Rice Bran Extract on the Preservation of Pork Burger Treated With High Pressure Processing.” Journal of Food Processing and Preservation 44, no. 1: e14313.

[fsn371537-bib-0055] Martillanes, S. , J. Rocha‐Pimienta , M. V. Gil , M. C. Ayuso‐Yuste , and J. Delgado‐Adamez . 2020. “Antioxidant and Antimicrobial Evaluation of Rice Bran ( *Oryza sativa* L.) Extracts in a Mayonnaise‐Type Emulsion.” Food Chemistry 308: 125633. 31644968 10.1016/j.foodchem.2019.125633

[fsn371537-bib-0056] Meng, Y. , and C. Li . 2021. “Conformational Changes and Functional Properties of Whey Protein Isolate‐Polyphenol Complexes Formed by Non‐Covalent Interaction.” Food Chemistry 364: 129622. 34175622 10.1016/j.foodchem.2021.129622

[fsn371537-bib-0057] Movaghar, S. , A. Pourfarzad , and A. Mehregan Nikoo . 2025. “Optimization of Brown Rice Protein Bars Formulated With Arabic and Persian Gums: Insights From Physicochemical, FTIR, and Chemometric Analyses.” LWT 231: 118303.

[fsn371537-bib-0058] Muley, A. B. , and R. S. Singhal . 2020. “Extension of Postharvest Shelf Life of Strawberries (Fragaria Ananassa) Using a Coating of Chitosan‐Whey Protein Isolate Conjugate.” Food Chemistry 329: 127213. 32516713 10.1016/j.foodchem.2020.127213

[fsn371537-bib-0059] Nemli, E. , G. Ozkan , B. Gultekin Subasi , et al. 2024. “Interactions Between Proteins and Phenolics: Effects of Food Processing on the Content and Digestibility of Phenolic Compounds.” Journal of the Science of Food and Agriculture 104, no. 5: 2535–2550. 38318731 10.1002/jsfa.13275

[fsn371537-bib-0060] Ngo, T. V. , S. Kusumawardani , K. Kunyanee , and N. Luangsakul . 2022. “Polyphenol‐Modified Starches and Their Applications in the Food Industry: Recent Updates and Future Directions.” Food 11: 3384. 10.3390/foods11213384 PMC9658643 36359996

[fsn371537-bib-0061] Nidhishree, A. , R. Menezes , V. Hillemane , and K. S. Bhat . 2024. “Rice Bran as a Sustainable Source for Value Added Materials: An Overview.” Discover Materials 4: 93.

[fsn371537-bib-0062] Okpala, C. O. R. , G. Bono , M. L. Geraci , G. Sardo , S. Vitale , and C. J. Schaschke . 2016. “Lipid Oxidation Kinetics of Ozone‐Processed Shrimp During Iced Storage Using Peroxide Value Measurements.” Food Bioscience 16: 5–10.

[fsn371537-bib-0063] Peanparkdee, M. , and S. Iwamoto . 2019. “Bioactive Compounds From By‐Products of Rice Cultivation and Rice Processing: Extraction and Application in the Food and Pharmaceutical Industries.” Trends in Food Science & Technology 86: 109–117.

[fsn371537-bib-0064] Peanparkdee, M. , J. Patrawart , and S. Iwamoto . 2019. “Effect of Extraction Conditions on Phenolic Content, Anthocyanin Content and Antioxidant Activity of Bran Extracts From Thai Rice Cultivars.” Journal of Cereal Science 86: 86–91.

[fsn371537-bib-0065] Peleg, M. 2019. “The Instrumental Texture Profile Analysis Revisited.” Journal of Texture Studies 50, no. 5: 362–368. 30714161 10.1111/jtxs.12392

[fsn371537-bib-0066] Pourfarzad, A. , Z. Ahmadian , and M. H. Tavassoli‐Kafrani . 2019. “The Effect of Sodium Stearoyl Lactylate on Structural Changes of Wheat Gluten in a Model System Fortified With Inulin: Investigation With Fourier Transform Infrared Spectroscopy.” Bioactive Carbohydrates and Dietary Fibre 17: 100175.

[fsn371537-bib-0067] Pourfarzad, A. , and R. Kisomi . 2020. “Effect of Lecithin and Mono‐and di‐Glyceride on Quality and Shelf Life of Hazelnut Butter: Chemometric Approach.” Polish Journal of Food and Nutrition Sciences 70, no. 4: 399–408.

[fsn371537-bib-0068] Pourfarzad, A. , M. B. H. Najafi , M. H. H. Khodaparast , and M. H. Khayyat . 2015. “Serish Inulin and Wheat Biopolymers Interactions in Model Systems as a Basis for Understanding the Impact of Inulin on Bread Properties: A FTIR Investigation.” Journal of Food Science and Technology 52, no. 12: 7964–7973. 26604368 10.1007/s13197-015-1939-4 PMC4648905

[fsn371537-bib-0069] Pourfarzad, A. , and N. Taleb Derakhshan . 2021. “Effect of Xanthan, Guar and Tragacanth on Quality and Shelf Life of Hazelnut Sauce: Study With Generalized Regression, PCA and PLSR Techniques.” Journal of Food Measurement and Characterization 15: 5008–5020.

[fsn371537-bib-0070] Pourfarzad, A. , and A. Yousefi . 2021. “Effect of Different Excipients on Physicochemical Properties of the Functional Rice Bran Tablet: Univariate and Multivariate Studies on a Novel Food Supplement.” Journal of Food Measurement and Characterization 15, no. 2: 1359–1369.

[fsn371537-bib-0071] Pourghasemian, P. , A. Pourfarzad , and A. Babakhani . 2025a. “Kinetic Modeling of Physicochemical Changes in Protein Bars Fortified With Spirulina and Phycocyanin During Storage.” Innovative Food Technologies 13, no. 1: 23–35.

[fsn371537-bib-0072] Pourghasemian, P. , A. Pourfarzad , and A. Babakhani . 2025b. “Optimization of a Brown Rice Protein Bar Supplemented With Spirulina and Phycocyanin: FTIR‐Based Analysis of Protein Secondary Structures and Multivariate Quality Assessment.” Applied Food Research 5, no. 2: 101259.

[fsn371537-bib-0073] Ruen‐Ngam, D. , C. Thawai , R. Nokkoul , and S. Sukonthamut . 2014. “Gamma‐Oryzanol Extraction From Upland Rice Bran.” International Journal of Bioscience, Biochemistry and Bioinformatics 4, no. 4: 252.

[fsn371537-bib-0074] Saleh, A. S. , P. Wang , N. Wang , L. Yang , and Z. Xiao . 2019. “Brown Rice Versus White Rice: Nutritional Quality, Potential Health Benefits, Development of Food Products, and Preservation Technologies.” Comprehensive Reviews in Food Science and Food Safety 18, no. 4: 1070–1096. 33336992 10.1111/1541-4337.12449

[fsn371537-bib-0075] Salitlertthanasin, P. 2017. “Product Development of Thai Rice Cereal (Khao‐Mao) Bar With Garlic Flavor and its Shelf‐life Using Accerelated Method.”

[fsn371537-bib-0076] Samuel, K. S. , and N. Peerkhan . 2020. “Pearl Millet Protein Bar: Nutritional, Organoleptic, Textural Characterization, and In‐Vitro Protein and Starch Digestibility.” Journal of Food Science and Technology 57, no. 9: 3467–3473. 32728293 10.1007/s13197-020-04381-x PMC7374522

[fsn371537-bib-0077] Seneviratne, K. P. , N. Anjali , C. M. Senanayake , N. Jayathilaka , and K. N. Seneviratne . 2022. “Ethanolic Extract of Rice Bran: A Thermally Stable Preservative for Edible Oils and Cake.” Food Production, Processing and Nutrition 4, no. 1: 14.

[fsn371537-bib-0078] Shahidi, F. , and C. S. Dissanayaka . 2023. “Phenolic‐Protein Interactions: Insight From In‐Silico Analyses–a Review.” Food Production, Processing and Nutrition 5, no. 1: 2.

[fsn371537-bib-0079] Showkat, S. , A. H. Dar , S. Khan , and M. Gani . 2018. “Effect of Mung Bean and Rice on Physico‐Chemical, Sensory and Microstructural Properties of Cereal Bars.” Carpathian Journal of Food Science and Technology 10, no. 4: 70–78.

[fsn371537-bib-0080] Spaggiari, M. , C. Dall'Asta , G. Galaverna , and M. D. del Castillo Bilbao . 2021. “Rice Bran By‐Product: From Valorization Strategies to Nutritional Perspectives.” Food 10, no. 1: 85. 10.3390/foods10010085 PMC7824317 33406743

[fsn371537-bib-0081] Srebernich, S. M. , G. M. S. Gonçalves , R. D. C. S. C. Ormenese , and C. R. G. Ruffi . 2016. “Physico‐Chemical, Sensory and Nutritional Characteristics of Cereal Bars With Addition of Acacia Gum, Inulin and Sorbitol.” Food Science and Technology 36: 555–562.

[fsn371537-bib-0082] Srisuk, N. , and S. Jirasatid . 2023. “Development of Instant Pumpkin‐Fingerroot Drink Powder and Its Shelf Life Modeling.” Life Sciences and Environment Journal 24, no. 1: 161–182.

[fsn371537-bib-0083] Sukhonthara, S. , K. Kaewka , and C. Theerakulkait . 2016. “Inhibitory Effect of Rice Bran Extracts and Its Phenolic Compounds on Polyphenol Oxidase Activity and Browning in Potato and Apple Puree.” Food Chemistry 190: 922–927. 26213057 10.1016/j.foodchem.2015.06.016

[fsn371537-bib-0084] Sunyoto, M. , R. Andoyo , and E. Masitoh . 2019. “Characteristics of High Protein Snack Bar Made of Modified Sweet Potato Flour.” IOP Conference Series: Earth and Environmental Science.

[fsn371537-bib-0085] Surarit, W. , C. Jansom , N. Lerdvuthisopon , S. Kongkham , and P. Hansakul . 2015. “Evaluation of Antioxidant Activities and Phenolic Subtype Contents of Ethanolic Bran Extracts of Thai Pigmented Rice Varieties Through Chemical and Cellular Assays.” International Journal of Food Science and Technology 50, no. 4: 990–998.

[fsn371537-bib-0086] Świeca, M. , D. Dziki , and U. Gawlik‐Dziki . 2017. “Starch and Protein Analysis of Wheat Bread Enriched With Phenolics‐Rich Sprouted Wheat Flour.” Food Chemistry 228: 643–648. 28317775 10.1016/j.foodchem.2017.02.052

[fsn371537-bib-0087] Szydłowska, A. , D. Zielińska , A. Łepecka , M. Trząskowska , K. Neffe‐Skocińska , and D. Kołożyn‐Krajewska . 2020. “Development of Functional High‐Protein Organic Bars With the Addition of Whey Protein Concentrate and Bioactive Ingredients.” Agriculture 10, no. 9: 390.

[fsn371537-bib-0088] Szydłowska, A. , D. Zielińska , M. Trząskowska , et al. 2022. “Development of Ready‐To‐Eat Organic Protein Snack Bars: Assessment of Selected Changes of Physicochemical Quality Parameters and Antioxidant Activity Changes During Storage.” Food 11, no. 22: 3631. 10.3390/foods11223631 PMC9689689 36429223

[fsn371537-bib-0089] Tabaraki, R. , and A. Nateghi . 2011. “Optimization of Ultrasonic‐Assisted Extraction of Natural Antioxidants From Rice Bran Using Response Surface Methodology.” Ultrasonics Sonochemistry 18, no. 6: 1279–1286. 21612968 10.1016/j.ultsonch.2011.05.004

[fsn371537-bib-0090] Talemi, F. P. , A. Pourfarzad , and S. Gheibi . 2025. “Synergistic Effects of Bee Pollen and Propolis Extract on Protein Bar Properties: A Multivariate Chemometric Analysis.” Discover Food 5, no. 1: 1–29.

[fsn371537-bib-0091] Tamime, A. , R. Robinson , P. Fellows , and D. Bender . 2018. “Woodhead Publishing Series in Food Science, Technology and Nutrition.” In Developing Food Products for Consumers With Specific Dietary Needs. Elsevier.

[fsn371537-bib-0092] Tan, B. L. , M. E. Norhaizan , and L. C. Chan . 2023. “Rice Bran: From Waste to Nutritious Food Ingredients.” Nutrients 15, no. 11: 2503. 37299466 10.3390/nu15112503 PMC10255292

[fsn371537-bib-0093] Tang, T. , R. Zhang , C. Chang , et al. 2025. “The Effects of Polyphenols on Texture and Flavor of Egg Yolk: A Molecular Docking Study.” Food 14, no. 2: 295. 10.3390/foods14020295 PMC11764978 39856960

[fsn371537-bib-0094] Tasnim, T. , P. C. Das , A. A. Begum , A. H. Nupur , and M. A. R. Mazumder . 2020. “Nutritional, Textural and Sensory Quality of Plain Cake Enriched With Rice Rinsed Water Treated Banana Blossom Flour.” Journal of Agriculture and Food Research 2: 100071.

[fsn371537-bib-0095] Thakur, M. , C. Sharma , A. Mehta , and D. D. Torrico . 2022. “Health Claim Effects on Consumer Acceptability, Emotional Responses, and Purchase Intent of Protein Bars.” Journal of Agriculture and Food Research 8: 100291.

[fsn371537-bib-0096] Theerakulkait, C. , and K. Boonsiripiphat . 2007. “Effects of Rice Bran Extract on Browning and Polyphenol Oxidase Activity in Vegetable and Fruit.” Agriculture and Natural Resources 41, no. 5: 272–278.

[fsn371537-bib-0097] Torres, E. , E. Castro , R. Santana , J. Cardoso , C. Soaresa , and Á. Lima . 2011. “Cereal Bar Development Using Exotic Fruit. Proceedings of 11th ICEF Conference on Engineering and Food, Food Process Engineering in a Changing World, Athens, Greece.”

[fsn371537-bib-0098] Trząskowska, M. , K. Neffe‐Skocińska , A. Okoń , et al. 2022. “Safety Assessment of Organic High‐Protein Bars During Storage at Ambient and Refrigerated Temperatures.” Applied Sciences 12, no. 17: 8454.

[fsn371537-bib-0099] Turgut, S. S. , E. Karacabey , and E. Küçüköner . 2014. “Potential of Image Analysis Based Systems in Food Quality Assessments and Classifications. Foodbalt 2014, 9th Baltic Conference on Food Science and Technology‐Conference Proceedings.”

[fsn371537-bib-0100] Tyl, C. , and G. D. Sadler . 2017. “pH and Titratable Acidity.” In Food Analysis, 389–406. Springer.

[fsn371537-bib-0101] Umme, H. , R. M. Ashadujjaman , H. M. Mehedi , T. M. Afroz , A. Delara , and M. M. A. Rahman . 2021. “Nutritional, Textural, and Sensory Quality of Bars Enriched With Banana Flour and Pumpkin Seed Flour.” Foods and Raw Materials 9, no. 2: 282–289.

[fsn371537-bib-0102] Usman, M. , M. Nakagawa , and S. Cheng . 2023. “Emerging Trends in Green Extraction Techniques for Bioactive Natural Products.” PRO 11, no. 12: 3444.

[fsn371537-bib-0103] Wanyo, P. , N. Meeso , and S. Siriamornpun . 2014. “Effects of Different Treatments on the Antioxidant Properties and Phenolic Compounds of Rice Bran and Rice Husk.” Food Chemistry 157: 457–463. 24679804 10.1016/j.foodchem.2014.02.061

[fsn371537-bib-0104] Wu, N. N. , B. Tan , S. S. Li , et al. 2017. “Cooking Quality, Antioxidant Properties, and Starch Digestibility of Wheat Noodles Substituted With Extruded Brown Rice Flour.” Cereal Chemistry 94, no. 3: 464–470.

[fsn371537-bib-0105] Xue, H. , J. Feng , Y. Tang , et al. 2024. “Research Progress on the Interaction of the Polyphenol–Protein–Polysaccharide Ternary Systems.” Chemical and Biological Technologies in Agriculture 11, no. 1: 95.

[fsn371537-bib-0106] Yadav, L. , and V. Bhatnagar . 2015. “Optimization of Ingredients in Cereal Bar.” Food Science Research Journal 6, no. 2: 273–278.

